# Genome-Wide Association Scan Meta-Analysis Identifies Three Loci Influencing Adiposity and Fat Distribution

**DOI:** 10.1371/journal.pgen.1000508

**Published:** 2009-06-26

**Authors:** Cecilia M. Lindgren, Iris M. Heid, Joshua C. Randall, Claudia Lamina, Valgerdur Steinthorsdottir, Lu Qi, Elizabeth K. Speliotes, Gudmar Thorleifsson, Cristen J. Willer, Blanca M. Herrera, Anne U. Jackson, Noha Lim, Paul Scheet, Nicole Soranzo, Najaf Amin, Yurii S. Aulchenko, John C. Chambers, Alexander Drong, Jian'an Luan, Helen N. Lyon, Fernando Rivadeneira, Serena Sanna, Nicholas J. Timpson, M. Carola Zillikens, Jing Hua Zhao, Peter Almgren, Stefania Bandinelli, Amanda J. Bennett, Richard N. Bergman, Lori L. Bonnycastle, Suzannah J. Bumpstead, Stephen J. Chanock, Lynn Cherkas, Peter Chines, Lachlan Coin, Cyrus Cooper, Gabriel Crawford, Angela Doering, Anna Dominiczak, Alex S. F. Doney, Shah Ebrahim, Paul Elliott, Michael R. Erdos, Karol Estrada, Luigi Ferrucci, Guido Fischer, Nita G. Forouhi, Christian Gieger, Harald Grallert, Christopher J. Groves, Scott Grundy, Candace Guiducci, David Hadley, Anders Hamsten, Aki S. Havulinna, Albert Hofman, Rolf Holle, John W. Holloway, Thomas Illig, Bo Isomaa, Leonie C. Jacobs, Karen Jameson, Pekka Jousilahti, Fredrik Karpe, Johanna Kuusisto, Jaana Laitinen, G. Mark Lathrop, Debbie A. Lawlor, Massimo Mangino, Wendy L. McArdle, Thomas Meitinger, Mario A. Morken, Andrew P. Morris, Patricia Munroe, Narisu Narisu, Anna Nordström, Peter Nordström, Ben A. Oostra, Colin N. A. Palmer, Felicity Payne, John F. Peden, Inga Prokopenko, Frida Renström, Aimo Ruokonen, Veikko Salomaa, Manjinder S. Sandhu, Laura J. Scott, Angelo Scuteri, Kaisa Silander, Kijoung Song, Xin Yuan, Heather M. Stringham, Amy J. Swift, Tiinamaija Tuomi, Manuela Uda, Peter Vollenweider, Gerard Waeber, Chris Wallace, G. Bragi Walters, Michael N. Weedon, The Wellcome Trust Case Control Consortium, Jacqueline C. M. Witteman, Cuilin Zhang, Weihua Zhang, Mark J. Caulfield, Francis S. Collins, George Davey Smith, Ian N. M. Day, Paul W. Franks, Andrew T. Hattersley, Frank B. Hu, Marjo-Riitta Jarvelin, Augustine Kong, Jaspal S. Kooner, Markku Laakso, Edward Lakatta, Vincent Mooser, Andrew D. Morris, Leena Peltonen, Nilesh J. Samani, Timothy D. Spector, David P. Strachan, Toshiko Tanaka, Jaakko Tuomilehto, André G. Uitterlinden, Cornelia M. van Duijn, Nicholas J. Wareham, Hugh Watkins for the PROCARDIS consortia, Dawn M. Waterworth, Michael Boehnke, Panos Deloukas, Leif Groop, David J. Hunter, Unnur Thorsteinsdottir, David Schlessinger, H.-Erich Wichmann, Timothy M. Frayling, Gonçalo R. Abecasis, Joel N. Hirschhorn, Ruth J. F. Loos, Kari Stefansson, Karen L. Mohlke, Inês Barroso, Mark I. McCarthy for the GIANT consortium

**Affiliations:** 1 Wellcome Trust Centre for Human Genetics, University of Oxford, , Oxford, United Kingdom; 2 Institute of Epidemiology, Helmholtz Zentrum München, National Research Center for Environment and Health, Neuherberg, Germany; 3 Institute of Epidemiology and Preventive Medicine, University of Regensburg, Regensburg, Germany; 4 Division of Genetic Epidemiology, Department of Medical Genetics, Molecular and Clinical Pharmacology, Innsbruck Medical University, Innsbruck, Austria; 5 deCODE Genetics, Reykjavik, Iceland; 6 Department of Nutrition, Harvard School of Public Health, Boston, Massachusetts, United States of America; 7 Channing Laboratory, Department of Medicine, Brigham and Women's Hospital, Boston, Massachusetts, United States of America; 8 Department of Gastroenterology, Massachusetts General Hospital, Harvard Medical School, Boston, Massachusetts, United States of America; 9 Metabolism Initiative and Program in Medical and Population Genetics, Broad Institute of MIT and Harvard, Boston, Massachusetts, United States of America; 10 Department of Biostatistics, Center for Statistical Genetics, University of Michigan, Ann Arbor, Michigan, United States of America; 11 Oxford Centre for Diabetes, Department of Endocrinology and Metabolism, University of Oxford, Oxford, United Kingdom; 12 Medical Genetics, Clinical Pharmacology and Discovery Medicine, King of Prussia, Pennsylvania, United States of America; 13 Center for Statistical Genetics, Department of Biostatistics, University of Michigan, Ann Arbor, Michigan, United States of America; 14 Department of Twin Research and Genetic Epidemiology, King's College London, London, United Kingdom; 15 Wellcome Trust Sanger Institute, Hinxton, Cambridge, United Kingdom; 16 Department of Epidemiology, Erasmus Medical Center, Rotterdam, The Netherlands; 17 Department of Epidemiology and Public Health, Imperial College London, London, United Kingdom; 18 MRC Epidemiology Unit, Institute of Metabolic Science, Addenbrooke's Hospital, Cambridge, United Kingdom; 19 Divisions of Genetics and Endocrinology, Program in Genomics, Children's Hospital, Boston, Massachusetts, United States of America; 20 Department of Internal Medicine, Erasmus Medical Center, Rotterdam, The Netherlands; 21 Istituto di Neurogenetica e Neurofarmacologia, Consiglio Nazionale delle Ricerche, Cagliari, Italy; 22 The MRC Centre for Causal Analyses in Translational Epidemiology, University of Bristol, Bristol, United Kingdom; 23 Department of Clinical Sciences, Diabetes, and Endocrinology Research Unit, University Hospital Malmö, Lund University, Malmö, Sweden; 24 Geriatric Unit, Azienda Sanitaria Firenze (ASF), Florence, Italy; 25 Physiology and Biophysics, University of Southern California School of Medicine, Los Angeles, California, United States of America; 26 National Human Genome Research Institute, Bethesda, Maryland, United States of America; 27 Pediatric Oncology Branch, Center for Cancer Research, National Cancer Institute, National Institutes of Health, Department of Health and Human Services, Bethesda, Maryland, United States of America; 28 Department of Epidemiology and Public Health, Imperial College London, London, United Kingdom; 29 MRC Epidemiology Resource Centre, University of Southampton, Southampton, United Kingdom; 30 Program in Medical and Population Genetics, Broad Institute of MIT and Harvard, Cambridge, Massachusetts, United States of America; 31 BHF Glasgow Cardiovascular Research Centre, University of Glasgow, Glasgow, United Kingdom; 32 Diabetes Research Group, Division of Medicine and Therapeutics, Ninewells Hospital and Medical School, University of Dundee, Dundee, United Kingdom; 33 Department of Social Medicine, University of Bristol, Bristol, United Kingdom; 34 Clinical Research Branch, National Institute on Aging, Baltimore, Maryland, United States of America; 35 Centre for Human Nutrition, University of Texas Southwestern Medical Centre, Dallas, Texas, United States of America; 36 Division of Community Health Sciences, St George's University of London, London, United Kingdom; 37 Atherosclerosis Research Unit, Department of Medicine, Karolinska Institutet, Stockholm, Sweden; 38 KTL-National Public Health Institute, Helsinki, Finland; 39 Division of Human Genetics, University of Southampton, Southampton, United Kingdom; 40 Folkhälsan Research Center, Malmska Municipal Health Center and Hospital, Jakobstad, Finland; 41 Department of Medicine, Helsinki University Central Hospital, University of Helsinki, Helsinki, Finland; 42 Finnish Institute of Occpational Health, Oulu, Finland; 43 Centre National de Genotypage, Evry, France; 44 The William Harvey Research Institute, Barts and The London School of Medicine and Dentistry, Queen Mary University of London, London, United Kingdom; 45 Department of Surgical and Perioperative Sciences, Section for Sports Medicine, Umeå University, Umeå, Sweden; 46 Department of Community Medicine and Rehabilitation, Section of Geriatrics, Umeå University Hospital, Umeå, Sweden; 47 Department of Clinical Genetics, Erasmus Medical Center, Rotterdam, The Netherlands; 48 Population Pharmacogenetics Group, Biomedical Research Centre, Ninewells Hospital and Medical School, University of Dundee, Dundee, United Kingdom; 49 Department of Cardiovascular Medicine, University of Oxford, Oxford, United Kingdom; 50 Genetic Epidemiology and Clinical Research Group, Department of Public Health and Clinical Medicine, Section for Medicine, Umeå University Hospital, Umeå, Sweden; 51 Department of Clinical Chemistry, University of Oulu, Oulu, Finland; 52 Department of Public Health and Primary Care, Institute of Public Health, University of Cambridge, Cambridge, United Kingdom; 53 Unita' Operativa Geriatrica, Instituto Nazionale Ricovero e Cura per Anziani (INRCA) IRCCS, Rome, Italy; 54 Department of Molecular Medicine, National Public Health Institute, Helsinki, Finland; 55 Research Program of Molecular Medicine, University of Helsinki, Helsinki, Finland; 56 Department of Medicine and Internal Medicine, Centre Hospitalier Universitaire Vaudois (CHUV), Lausanne, Switzerland; 57 Genetics of Complex Traits, Institute of Biomedical and Clinical Science, Peninsula Medical School, Exeter, United Kingdom; 58 Division of Epidemiology, Statistics, and Prevention Research, National Institute of Child Health and Human Development, Bethesda, Maryland, United States of America; 59 Ealing Hospital, Ealing Hospital National Health Service Trust, Southall, London, United Kingdom; 60 Bristol Genetic Epidemiology Laboratories, Department of Social Medicine, University of Bristol, Bristol, United Kingdom; 61 Department of Public Health and Clinical Medicine, Section for Nutritional Research (Umeå Medical Biobank), Umeå University, Umeå, Sweden; 62 Institute of Health Sciences, University of Oulu, Biocenter Oulu, University of Oulu, Oulu, Finland; 63 Department of Child and Adolescent Health, National Public Health Institute, Oulu, Finland; 64 National Heart and Lung Institute, Imperial College London Hammersmith Hospital, London, United Kingdom; 65 Gerontology Research Center, National Institute on Aging, Baltimore, Maryland, United States of Ameica; 66 Department of Cardiovascular Sciences, University of Leicester, Glenfield Hospital, Leicester, United Kingdom; 67 Medstar Research Institute, Baltimore, Maryland, United States of America; 68 Diabetes Unit, Department of Epidemiology and Health Promotion, National Public Health Institute, Helsinki, Finland; 69 Institute of Metabolic Science, Addenbrookes Hospital, Cambridge, United Kingdom; 70 Program in Molecular and Genetic Epidemiology, Harvard School of Public Health, Boston, Massachusetts, United States of America; 71 Broad Institute of MIT and Harvard, Boston, Massachusetts, United States of America; 72 Faculty of Medicine, University of Iceland, Reykjavík, Iceland; 73 Department of Genetics, Harvard Medical School, Boston, Massachusetts, United States of America; 74 Department of Genetics, University of North Carolina, Chapel Hill, North Carolina, United States of America; 75 National Institute for Health Research, Oxford Biomedical Research Centre, University of Oxford, Oxford, United Kingdom; University of Alabama at Birmingham, United States of America

## Abstract

To identify genetic loci influencing central obesity and fat distribution, we performed a meta-analysis of 16 genome-wide association studies (GWAS, N = 38,580) informative for adult waist circumference (WC) and waist–hip ratio (WHR). We selected 26 SNPs for follow-up, for which the evidence of association with measures of central adiposity (WC and/or WHR) was strong and disproportionate to that for overall adiposity or height. Follow-up studies in a maximum of 70,689 individuals identified two loci strongly associated with measures of central adiposity; these map near *TFAP2B* (WC, *P* = 1.9×10^−11^) and *MSRA* (WC, *P* = 8.9×10^−9^). A third locus, near *LYPLAL1*, was associated with WHR in women only (*P* = 2.6×10^−8^). The variants near *TFAP2B* appear to influence central adiposity through an effect on overall obesity/fat-mass, whereas *LYPLAL1* displays a strong female-only association with fat distribution. By focusing on anthropometric measures of central obesity and fat distribution, we have identified three loci implicated in the regulation of human adiposity.

## Author Summary

Here, we describe a meta-analysis of genome-wide association data from 38,580 individuals, followed by large-scale replication (in up to 70,689 individuals) designed to uncover variants influencing anthropometric measures of central obesity and fat distribution, namely waist circumference (WC) and waist–hip ratio (WHR). This work complements parallel efforts that have been successful in defining variants impacting overall adiposity and focuses on the visceral fat accumulation which has particularly strong relationships to metabolic and cardiovascular disease. Our analyses have identified two loci (*TFAP2B* and *MSRA*) associated with WC, and a further locus, near *LYPLAL1*, which shows gender-specific relationships with WHR (all to levels of genome-wide significance). These loci vary in the strength of their associations with overall adiposity, and *LYPLAL1* in particular appears to have a specific effect on patterns of fat distribution. All in all, these three loci provide novel insights into human physiology and the development of obesity.

## Introduction

The accumulation of abnormal amounts of intra-abdominal fat (central adiposity) is associated with serious adverse metabolic and cardiovascular outcomes, including type 2 diabetes (T2D) and atherosclerotic heart disease [1]. Indeed, because the medical consequences of increasing fat mass are disproportionately attributable to the extent of central adiposity, measures of overall adiposity, such as body mass index (BMI), fail to capture all of this risk [2],[3].

Measures of central and overall adiposity are highly correlated (BMI has r^2^∼0.9 with waist circumference [WC] and ∼0.6 with waist-hip ratio [WHR], Table S1). WC and WHR are correlated with more precise measures of intra-abdominal fat measured by MRI in obese women (r^2^∼0.6 and 0.5, respectively) [4]. Several lines of evidence indicate that individual variability in patterns of fat distribution involves local, depot-specific processes, which are independent of the predominantly neuronal mechanisms that control overall energy balance. First, anthropometric measures of central adiposity are highly heritable [5] and, after correcting for BMI, heritability estimates remain high (∼60% for WC and ∼45% for WHR) [6]. Second, there are substantial gender-specific differences in fat distribution, and these appear to reflect genetic influences [7]. Third, uncommon monogenic syndromes (the partial lipodystrophies) demonstrate that DNA variants can have dramatic effects on the development and/or maintenance of specific regional fat-depots [8].

Efforts to identify common and rare variants influencing BMI and risk of obesity have emphasized the key role of neuronal (hypothalamic) regulation of overall adiposity [9]–[17] but provided few clues to processes that are specifically responsible for individual variation in central obesity and fat distribution. Definition of the mechanisms involved in the regulation of fat distribution in general, and visceral fat mass in particular, is therefore key to understanding obesity and its accompanying morbidity and mortality. Given the challenges associated with the pharmacological manipulation of hypothalamic processes, the identification of pathways influencing abdominal fat accumulation would also present novel opportunities for therapeutic development.

With this in mind, we set out to identify genetic loci influencing anthropometric measures of central obesity and fat distribution, namely, WC and WHR. Our meta-analysis of 16 genome wide association studies (GWAS), followed by large-scale replication testing, generating a combined sample of up to 118,691 individuals of European origin, has identified three loci associated with these critical biomedical traits.

## Results/Discussion

**Figure 1:**
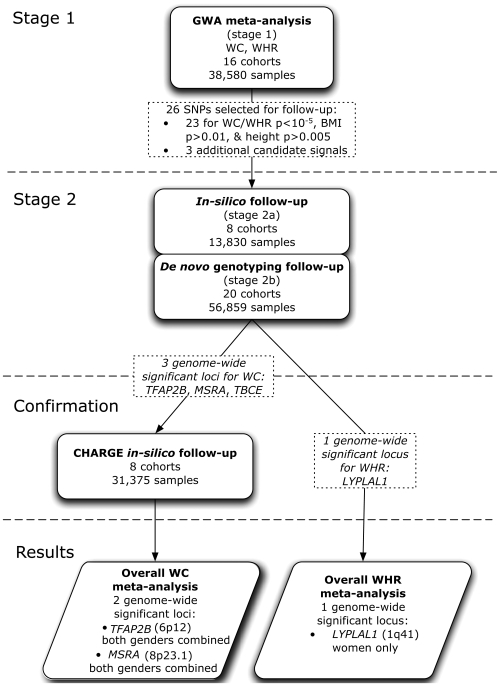
Project outline. We started out with a meta-analysis of GWAS data from 16 cohorts comprising 38,580 individuals informative for WC and 37,670 for WHR. We selected 23 SNPs of our top signals based on the following criteria (Table S2): preliminary stage 1 meta-analysis P-value≤10^−5^, BMI P-value>0.01 and height P-value>5×10^−3^. We supplemented these 23 independent loci (r^2^<0.2) SNPs with three additional candidate signals. Further, we excluded recently reported BMI loci (Table S10) [10], [14], [16]–[17]. These 26 SNPs were followed up in our stage 2 samples (N = maximum of 70,689 individuals). Further, we sought to confirm WC signals reaching genome wide significance in the combined analysis of stage 1 and 2 data in GWA data from the CHARGE consortium (for which WHR was not available). The data from the Rotterdam and ERF cohorts (up to 6,702 individuals) which were included in both CHARGE and stage 2 data, were counted only once in the overall analysis.

Our strategy for identifying common variants influencing central adiposity is summarized in Figure 1. The study was based on an initial (“stage 1”) meta-analysis of GWAS data to identify SNPs strongly-associated with measures of central adiposity (see Table S2). We then focused our “stage 2” follow-up efforts on the subset of those signals for which the strength of the evidence of association for measures of central adiposity (WC and WHR) appeared to be substantially stronger than that observed for overall adiposity and/or height. We reasoned that this subset of signals would be enriched for variants with preferential influences on central fat accumulation.

### GWAS Meta-Analysis for Anthropometric Measures of Central Obesity

The stage 1 meta-analysis combined data from 16 GWAS scans (N = 38,580, all of European ancestry) informative for anthropometric phenotypes (Table S3). We selected two complementary but related measures of central adiposity for analysis: waist circumference (WC) and waist-hip ratio (WHR) (Table S4). In total, 2,573,738 directly typed or imputed SNPs were tested for association using regression analysis under an additive model (see Table S5 for details). We conducted a weighted Z-score meta-analysis combining gender- and sample-specific association P-values gathered from each contributing study. We also performed an inverse-variance meta-analysis using regression results (β-estimates and standard errors) after applying uniform analysis procedures across all contributing samples. The results of the two meta-analyses were highly-congruent. Here, we report association P-values based on the former, as it was the first-completed and was used to select SNPs for follow-up genotyping. Reported effect-size estimates derive from the latter (see Methods for further details).

**Figure 2:**
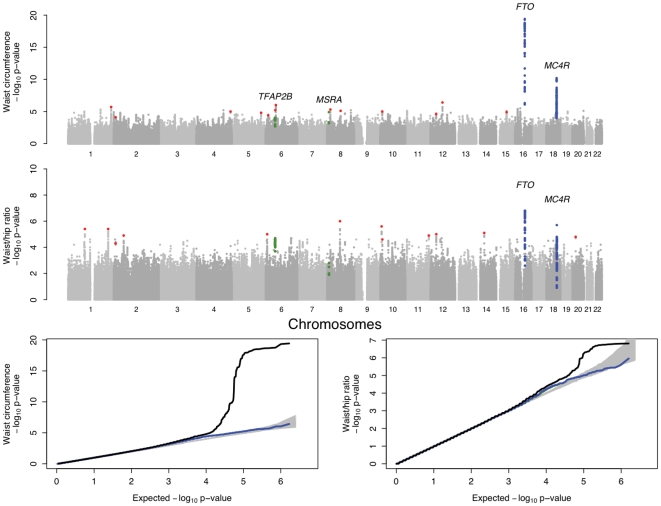
Genome-wide association results for GIANT (Stage 1). A. Manhattan plots showing significance of association of all SNPs in the Stage 1 GIANT meta-analysis with central obesity phenotypes. SNPs are plotted on the x-axis according to their position on each chromosome against association with central obesity measure (WC or WHR) on the y-axis (shown as −log10 P-value). SNPs that have been previously reported to show association with BMI is shown in blue [10],[14],[16] and the two regions showing strong associations in the overall, non-gender-stratified analyses are shown in green. Other SNPs taken forward into stage 2 follow-up are indicated in red. B. Quantile-quantile (QQ) plots of SNPs; after Stage 1 GIANT meta-analysis (black) and after removing any SNPs surrounding the recently reported BMI loci [10], [14], [16]–[17] (blue). The grey areas in the QQ plots represent the 95% confidence intervals around the test statistics and after excluding the recently reported BMI loci [10], [14], [16]–[17], there is no indication of excess of signal.

The individual studies as well as the results from the overall meta-analysis were corrected for residual inflation of the test statistic using genomic control methods [18]. The overall genomic control lambda (λ_GC_) in the mixed-gender analysis were λ_GC_WC_ = 1.09 (λ_GC_WC_1000_ = 1.003 [standardised to a sample size of 1000]) and λ_GC_WHR_ = 1.07 (λ_GC_WC_1000_ = 1.002) (see Text S1) [19]. From these data, we identified a set of 76 SNPs (one per independent region of association, based on an arbitrary follow-up P-value threshold of 10^−5^ in preliminary pre-GC corrected analyses) that showed evidence of association to one or both of the traits (Figure 2). As might have been expected given the strong correlations between measures of central adiposity and BMI, the most significant associations for WC and WHR were observed for SNPs mapping near *FTO* (rs1421085, WC, P = 3.7×10^−20^) and *MC4R* (rs17700144, WC, P = 6.2×10^−11^). These two markers are highly correlated (r^2^>0.8) with markers that represent two of the strongest signals for overall adiposity (Table S10) [9]–[10], [12]–[14], [16]–[17],[20].

### *In Silico* and *De Novo* Follow-Up

From this initial set of 76 WC- and/or WHR- associated signals, we sought to enrich for variants with specific impacts on central adiposity, by identifying a subset of 23 SNPs for which there was greatest evidence for a disproportionate effect on central adiposity, as opposed to overall adiposity or height. These 23 variants all had strong (i.e. P≤10^−5^) associations with WC and/or WHR while displaying only weak evidence of an association with overall adiposity (BMI, P>0.01) or adult height (P≥0.005) in the stage 1 GWAS meta-analysis data (Table S2). We also included three variants for reasons of biological candidacy, even though they did not precisely meet all P-value threshold criteria (see Table S2). Given the stage 1 sample size of 38,580, the follow-up P-value threshold of 10^−5^ provides 80% power to detect a per-allele beta of 0.045 (equivalent, for example, to a per-allele effect on WC of approximately 0.5 cm), given an additive model and MAF of 20%.

For these 26 SNPs, we obtained *in silico* follow-up data from another 8 studies with GWAS data (Stage 2a: maximum N = 13,830 individuals, all European-ancestry), and performed *de novo* genotyping in subjects from 20 additional studies (Stage 2b: maximum N = 56,859, all European-ancestry) (Table S3). Follow-up analyses were restricted to the precise phenotype(s) (WC and/or WHR) for which the SNP had been selected in stage 1 making a total of 30 SNP-phenotype combinations (Tables S2 and S6).

**Figure 3:**
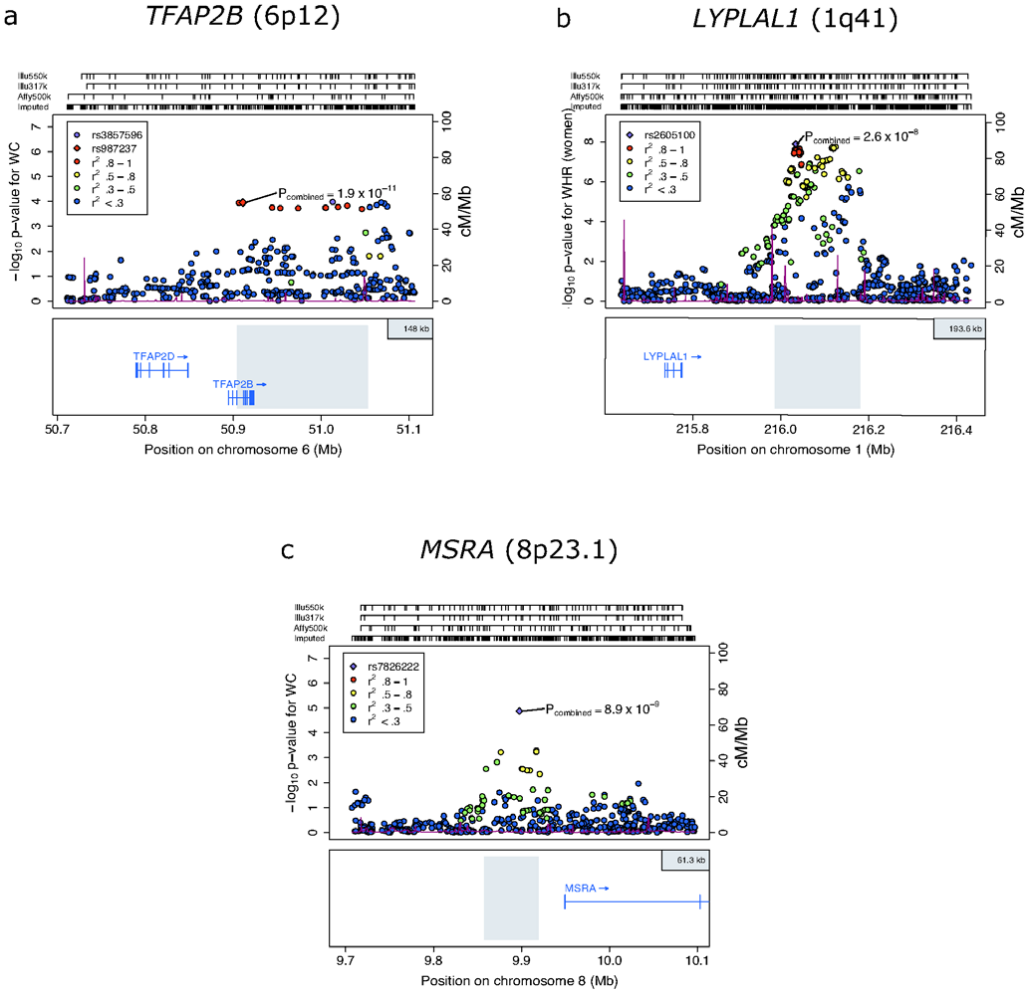
Regional plots of loci highlighted in this study. SNPs are plotted by position on chromosome against association (−log10 p-value) with central obesity phenotype (WC or WHR) using stage 1 (GWAS meta-analysis) data. In the case of panel (b), analyses are restricted to women only. In each panel, the SNP with the strongest association based on stage 1 data is denoted with a purple diamond: the P-value attached represents the final P-value attained across all available data (Table 1). Estimated recombination rates (from HapMap-CEU) are plotted in purple to reflect the local LD structure on a secondary Y-axis. The SNPs surrounding the most significant SNP (purple diamond) are color-coded (see inset) to reflect their LD with this SNP (using pair-wise r^2^ values from HapMap CEU). Genes and the position of exons, as well as the direction of transcription, are shown below the plots (using data from the UCSC genome browser, genome.ucsc.edu). The grey area marks the extent of the region that includes any SNP with r^2^≥0.3 relative to the SNP with the strongest stage 1 association signal. Hash marks represent SNP positions on each genotyping array used by any individual study and also show SNP positions after imputation.

After combining gender- and study-specific measures of association across all studies (maximum possible N = 109,269: Tables S2 and S3), we identified three signals reaching genome-wide levels of significance in the joint analysis of stage 1 and stage 2 data (P<5×10^−8^, Table 1, Figure 3). In all three instances, the association was observed with WC. The first (rs987237, chromosome 6p12: P = 4.5×10^−9^) maps near *TFAP2B*, which encodes transcription factor activating enhancer-binding protein 2 beta. The second (rs7826222, chromosome 8p23.1: P = 1.2×10^−8^) resides near *MSRA*, encoding methionine sulfoxide reductase A, whilst the third (rs6429082, chromosome 1q42.3: P = 2.6×10^−8^) is located within the *TBCE* (tubulin folding cofactor E) gene region.

**Table 1:** SNPs with genome-wide significant evidence for association with central adiposity and fat distribution.

Locus (Chromosomal Region)	SNP (Effect/Non-Effect)	Effect allele frequency (EAF) %	Phenotype	Gender	Stage	N*	β (SE)	Z-score	P-value**
					Stage 1	38,635	0.038 (0.010)	3.87	1.10×10^−4^
					Stage 2a	12,369	0.019 (0.017)	1.13	2.57×10^−1^
*TFAP2B* (6p12)	rs987237	16.4%	WC	both	Stage 2b	43,016	0.037 (0.009)	4.24	2.22×10^−5^
	(G/A)				Stage 1+2 Combined	94,021	0.035 (0.006)	5.86	4.54×10^−9^
					CHARGE	31,372	-	3.57	3.64×10^−4^
					**Overall**	118,691	-	6.72	1.87×10^−11^
					Stage 1	36,865	0.045 (0.011)	4.36	1.32×10^−5^
					Stage 2a	3,406†	0.023 (0.033)	0.73	4.63×10^−1^
*MSRA* (8p23.1)	rs7826222	18.3%	WC	both	Stage 2b	31,841	0.036 (0.011)	3.47	5.31×10^−4^
	(G/C)				Stage 1+2 Combined	72,113	0.040 (0.007)	5.7	1.20×10^−8^
					CHARGE	8,097†	-	1.09	2.76×10^−1^
					**Overall**	80,210	-	5.75	8.89×10^−9^
					Stage 1	21,397	0.062 (0.011)	5.69	1.30×10^−8^
					Stage 2a	6,021	0.035 (0.019)	1.74	8.17×10^−2^
*LYPLAL1* (1q41)	rs2605100	69.2%	WHR	women	Stage 2b	20,213	0.018 (0.011)	1.69	9.06×10^−2^
	(G/A)				**Stage 1+2 Combined**	47,633	0.040 (0.007)	5.57	2.55×10^−8^
					CHARGE	-	-	-	-
					Overall	-	-	-	-

Alleles: the first allele listed in the parenthesis is the effect allele, for which the allele frequency is given.

Stage 1: data from stage 1 GIANT GWAS meta-analysis.

Stage 2a: data from meta-analysis of *in silico* studies.

Stage 2b: data from meta-analysis of *de novo* genotyped studies.

Stage 1+2: data from stage 1 GIANT analyzed with *in silico* studies (Stage 2a) and *de novo* genotyped studies (Stage 2b).

Overall: data from meta-analysis of Stage1, 2a, 2b and CHARGE.

*: Total sample sizes do not always reflect the sum of component studies due to (a) rounding errors in non-integeric sample sizes arising from the weighting procedure; (b) overlap, for some markers, of data from the Rotterdam and ERF cohorts (up to 6,702 individuals) which were included in both CHARGE and stage 2 data, but which are counted only once in the overall analysis.

**: P-values: all P-values we report here are two-sided.

**†:** For the *MRSA* locus, genotypes for rs7826222 were only available for a subset of the CHARGE samples (N = 8,097). This is likely due to the fact that this SNP has been renamed to rs545854 in NCBI build 36 and was consequently one of the SNPs omitted from HapMap release 22 and therefore is not present in build 36 imputations based on that release of HapMap.

- Data not available. Effect size estimates and overall WHR results are not available as CHARGE only analysed WC using the weighted Z-score method (see Methods).

### Confirmation in CHARGE Consortium GWAS Data

As a final stage of confirmation, we analysed genotype data for rs987237, rs7826222 and rs6429082 made available to us by the CHARGE consortium, whose members had recently completed a GWAS meta-analysis of WC in 31,375 individuals (of which up to 6,702 individuals were overlapping with samples from our stage 2 and were removed before the joint meta-analysis).

At *TFAP2B*, CHARGE analyses revealed directionally-consistent association with WC (rs987237, N = 31,372, P = 3.6×10^−4^) resulting in a combined P-value of 1.9×10^−11^ (N = 118,691). At *MSRA*, genotypes for rs7826222 could only be imputed in a subset (N = 8,097) of CHARGE samples (this reflects SNP nomenclature issues rather than data quality – see Text S1). Nonetheless, the effect in CHARGE was directionally consistent (P = 0.28), and in the overall results (N = 80,210) for this SNP, the evidence for association with WC was improved (P = 8.9×10^−9^) (Table 1).

In contrast, rs6429082 in *TBCE* showed no evidence of association with WC in the full CHARGE data set (N = 31,373, P = 0.12). Since analysis of the combined data set no longer reached genome-wide significance (P = 2.9×10^−7^), further studies will be required to establish the status of this signal. For the *TFAP2B* and *MRSA* loci, there was no evidence of heterogeneity of effect size across the various sample sets, and no evidence that the inclusion of diabetes or coronary artery disease case samples had any impact on the associations (Table S2).

### Gender-Specific Association Analyses

Given the clear gender dimorphism of central obesity, and evidence that some genetic effects on fat distribution may be gender-specific [7], we reanalysed the stage 1 GWAS data, looking for effects restricted to males or females only. These analyses revealed a further locus of interest. SNPs, including rs2605100, within a gene desert on chromosome 1q41 (138 kb from *ZC3H11B* and 259 kb from *LYPLAL1*, encoding lysophospholipase-like protein 1) had shown modest evidence for association with WHR in our primary (both genders included) analysis (P = 3.6×10^−6^) (Table S2). However, in gender-specific analyses, this association was clearly restricted to females (P = 1.3×10^−8^; males: P = 0.50). When stage 1 and stage 2 data were combined, the female-only signal remained highly-significant (P = 2.6×10^−8^) (Table 1) with evidence of effect-size heterogeneity between genders (P = 1.1×10^−3^). As the CHARGE GWAS analyses were restricted to WC, we were unable to follow-up the *LYPLAL1* signal in these data.

### Disentangling Effects on Overall and Central Adiposity

We had designed this study to be complementary to equivalent analyses of overall adiposity (as measured by BMI) conducted on many of the same samples [10], [12]–[14],[16],[17]. By focusing on widely-available anthropometric proxies of central adiposity, and targeting replication to those signals which, in the GWAS data, had the most compelling evidence for disproportionate effects on central adiposity, our aim had been to enrich for variants influencing regional rather than overall obesity, and thereby overcome the very strong correlations between these measures.

We were interested therefore in establishing the extent to which the confirmed, genome-wide associations identified at/near *TFAP2B*, *MSRA* and *LYPLAL1* were indeed specific for central fat accumulation as opposed to being driven by other highly-correlated anthropometric traits (most notably overall adiposity as measured by BMI). To evaluate this, we used data from the stage 2 replication samples, from which we can expect to obtain less biased estimates of the relative effects across anthropometric phenotypes.

In the case of *TFAP2B*, these stage 2 data indicated that, notwithstanding the evidence for discordant effects in the stage 1 data (which led to its selection for follow up), rs987237 showed strong associations with overall adiposity (P = 7.0×10^−12^ for BMI in stage 2 alone). The association with WC remained only nominally significant in stage 2 (P = 0.02) after adjustment for BMI. The *TFAP2B* rs987237 G allele was weakly associated with overall fat mass (0.15% difference per-allele [P = 0.02] in 29,316 individuals with bioimpedance data; 0.25% difference per-allele [P = 0.02] in 13,039 additional individuals with dual energy X-ray absorptiometry (DXA) measures: Table S7). In the 7,346 individuals for which we had DXA information on fat distribution, there was no apparent association with percent central fat mass (P = 0.98), although this analysis is underpowered.

These data suggest that the chromosome 6p12 signal exerts its predominant effect on fat accumulation at multiple sites, a finding consistent with the known biology of *TFAP2B*, which is the most obvious candidate gene in the locus. *TFAP2B* encodes a transcription factor preferentially expressed in adipose tissue, and over-expression of the transcript in 3T3L1-adipocytes leads to insulin sensitivity via enhanced glucose transport and increased lipid accumulation [21],[22]. Over-expression of *TFAP2B* also down-regulates expression of the insulin-sensitizing hormone adiponectin by direct transcriptional repression [23]. Genetic variants within *TFAP2B* have recently been reported to correlate positively with *TFAP2B* transcript levels in adipose tissue [24]. Thus, *TFAP2B* can be added to the growing list of loci influencing overall adiposity [10],[14],[16],[17]. However, in contrast to most of the variants previously implicated in monogenic or multifactorial forms of obesity, which exert their effects on overall adiposity at the hypothalamic level [10], [12]–[14], [16]–[17], *TFAP2B* may be involved in global adipocyte response to positive energy balance.

In contrast, the signal on chromosome 1q41 (near *LYPLAL1*) showed relatively modest associations with overall obesity (stage 2, women only, P = 1.9×10^−4^ for BMI) and WC (P = 0.01). Crucially, the strength of the association with WHR was substantially greater after adjustment for BMI (stage 2, women only, P = 4.3×10^−6^). In the limited subset of women (N = 7,228) for whom direct measures of hip circumference (HC) could be retrieved, and in whom there was a proportionate signal for WHR (P = 5.2×10^−4^), we found no association with HC (P = 0.7) and a directionally consistent trend of association to WC (P = 0.06). Whilst these data would suggest that the *LYPLAL1* signal does indeed have a specific effect on fat distribution, our own DXA data on regional fat distribution are non-contributory (N = 5,455) (Table S7), and large-scale clinical imaging studies will be required to explore this further. The obvious candidate within this locus (although it lies ∼259 kb downstream of the most strongly-associated variant) is *LYPLAL1*. This gene encodes a lysophospholipase-like 1 protein thought to act as a triglyceride lipase and reported to be up-regulated in subcutaneous adipose tissue of obese subjects [25].

Biological connections between the *MSRA* locus and adiposity phenotypes are unclear at this stage. The signal near *MSRA* showed only weak association with overall adiposity (P = 2.2×10^−3^ for BMI in stage 2), but the strong association with WC in stage 2 samples became non-significant after BMI-adjustment (P = 0.11). The main proposed function of *MSRA* is to repair oxidative damage to proteins by enzymatic reduction of methionine sulfoxide. An alternative candidate in the vicinity is *TNKS*, which encodes a TRF1-interacting ankyrin-related ADP-ribose polymerase (tankyrase). Tankyrase is a peripheral membrane protein known to interact with insulin-responsive aminopeptidase (IRAP) in GLUT4 vesicles in adipocytes [26],[27]. Thus *TNKS* has a putative role in insulin-regulated glucose disposal into fat and other tissues.

### Variance Explained by the Associated Loci

We estimated the variance in these traits attributable to the loci discovered using data from the KORA-S4 sample (the largest population-based sample within stage 2). The explained variance of WC was estimated to be 0.05% for rs987237 (*TFAP2B*) and 0.04% for rs7826222 (*MSRA*). This corresponds to absolute WC effect sizes of 0.49 and 0.43 cm respectively (as estimated across all population based samples in stage 2). The SNP near *LYPLAL1* (rs2605100) explains 0.02% of the WHR variance in women (absolute effect size on WHR of 0.0014).

### Associations with Adverse Health Consequences

The accumulation of central adiposity has serious adverse health consequences including hyperlipidemia and increased risks of T2D. We examined the relationships between adiposity-related SNPs and these clinical phenotypes using available GWAS meta-analysis data (Text S1). We found an association between the WHR-increasing G-allele of rs2605100 (*LYPLAL1*) and increased fasting triglycerides (P = 3.9×10^−4^; Table S8) in data from a recent GWAS meta-analysis of 14,343 European samples [28]. This is further supported by a parallel GWAS meta-analysis effort in 19,840 samples where the G allele is similarly associated with increased triglycerides (P = 0.02) [29]. Using T2D case-control data from the DIAGRAM consortium [30], we found directionally-consistent, though only weak, associations with T2D-risk, most obviously at *TFAP2B* (P = 0.09; Table S9). An association between other non-HapMap *TFAP2B* variants and T2D has previously been reported in Japanese samples [21]. These T2D-associated variants show modest linkage disequilibrium to our WC associated SNP in UK samples (IVS1774_G/T and rs987237, r^2^ = 0.42; intron_1+2093_(A/C) and rs987237, r^2^ = 0.67). Thus, we see some evidence that the variants identified have anticipated effects on downstream phenotypes, although, as recently demonstrated for *FTO* (which has more marked effects on adiposity than the signals described here), analyses of this type have only limited power even in extremely large data sets [31].

In summary, by focusing on anthropometric measures of central obesity, we have identified three loci strongly implicated in the regulation of human adiposity and fat distribution. The extent of phenotypic variation explained by these variants is small. However, the variant or variants at each locus which are directly involved in influencing these traits are yet to be identified, and these may have more substantial effects. Even if this is not the case, effect size has very little bearing on the biological pertinence of these findings nor the potential impact of perturbing these pathways through therapeutic modification. Although determination of the influence of these signals on the development and maintenance of specific fat depots will require analyses that relate genetic variation to detailed imaging data in large numbers of subjects, the loci identified appear to highlight a variety of novel mechanisms involved in the regulation of adiposity. At this stage, it is unclear to what extent these same loci influence fat distribution in other ethnic groups, such as South Asians, in which patterns of fat distribution, and the relationships between fat distribution and metabolic disturbance, differ from those in Europeans. The data are consistent with a model whereby fat mass and distribution are determined through the concerted action of processes acting at the level of both the hypothalamus and peripheral fat depots.

## Methods

### Study Design Summary

Our study began with a genome-wide screen for discovery of loci potentially associated with two different anthropometric measures of central adiposity: waist circumference (WC) and waist-hip-ratio (WHR) [1]. For each of the traits we combined the summary statistics of 16 genome-wide association studies (GWAS) in meta-analyses with 38,580 (WC) and 37,670 (WHR) individuals, respectively (stage 1). These studies included nine population-based cohorts, four case cohorts (three for T2D and one for Hypertension), and three control cohorts (two originally paired with T2D and one with Breast Cancer) (Table S3). Following the discovery GWA meta-analysis, follow-up of our top association signals involved: (a) addition of data for markers of interest from studies with pre-existing “*in-silico*” GWA results (stage 2a; eight cohorts, maximum 13,830 individuals) and (b) “*de novo*” genotyping (stage 2b; 20 cohorts, maximum 56,859 individuals) giving a total of 70,689 (WC) or 61,612 (WHR) follow-up samples (collectively referred to as stage 2). In addition, genome wide signals for WC identified after stage 2 were confirmed using data with The Cohorts for Heart and Aging Research in Genomic Epidemiology (CHARGE) consortium, whose meta-analysis included eight studies totaling 31,375 individuals. All samples included in these analyses were of European ancestry. We also undertook gender specific analysis of the stage 1 GWAS. An overview of the study design and results is given in Figure 1.

### Genome-Wide Association Meta-Analysis (Stage 1)

#### Studies and phenotypes

All samples in the discovery cohorts were of European ancestry and detailed information on each of these studies is provided in Tables S3 and S4. Although we do not have specific age cut-offs for the individual cohorts the study participants are all adult (mean age between all cohorts = 55.7 years, range 31–70.3 years). All individuals provided informed consent and all studies were approved by local ethics committees.

#### Choice of phenotypes

The most appropriate adiposity measurements for assessing various fat compartments and the risk of adverse health outcomes is debated. Despite the close correlation between WC and BMI (Table S1), WC has been reported to have a BMI-independent impact on risk of death [1]. WHR is less strongly correlated to BMI than WC (Table S1) and is used as a more specific surrogate for fat distribution [1]. In our largest population based samples we see (as expected) that measures of central and overall adiposity are highly-correlated (BMI has r^2^∼0.9 with WC and ∼0.6 WHR, Table S1). When compared to the gold standard of MRI measures of central adiposity, WC and WHR are equally well-correlated to central adiposity (r^2^∼0.6, and 0.5, respectively) as are measures involving DXA (r^2^∼0.6) [4].

WC and WHR were measured in the individual cohorts using standard protocols. Recently a multi-centre comparison of WC measurements at different sites showed that the measurement at different centres have no substantial influence on the association to various adverse health outcomes [32]. In line with this we detect little, if any, heterogeneity at our significant associations, which indicates that it is unlikely that any differences in measurement protocols are having a substantial effect on these (Table S2).

#### Genotypes and imputation

Operational details of each of the 16 GWAS (including genotyping platforms, quality control filters for individuals and SNPs, and imputation and data analysis methods) can be found in Table S5. In summary, stage 1 genotypes were derived using different genotyping platforms; Affymetrix 500 k, Illumina HumanHap 550, Illumina HumanCNV-370DUO, Illumina HumanHap300 Duo Infinium, or Illumina HumanHap 300. To obtain a marker set that was common to all studies, and to increase overall coverage of the whole genome, we imputed all SNPs reported in the CEU sample in HapMap Phase II using imputation algorithms, yielding a maximum of 2,573,738 million SNPs available for analysis in one or more studies. Imputations were performed after excluding samples and SNPs that did not meet the study-specific quality control criteria. Genotypes were imputed for SNPs not present in the genome-wide arrays or for those where genotyping had failed to meet the QC criteria. We used either of two different software packages for the imputation: MACH [33] (http://www.sph.umich.edu/csg/abecasis/MACH/index.html) and IMPUTE [34] (http://www.stats.ox.ac.uk/~marchini/software).

Quality control (QC) criteria for SNPs to be included in the meta-analysis were minor allele frequency (MAF)≥1% and for imputed SNPs good imputation quality, which was defined as proper_info≥0.4 (for studies analysed with IMPUTE) or rsq-hat≥0.3 (for studies analysed using MACH). The rsq_hat measure allows us to assess imputation accuracy for markers with many different allele frequencies. In comparison to filters based on the accuracy of individual genotype calls, it generally translates into a more stringent standard for rare SNPs and a more lenient standard for common SNPs. For intuition on how the measure performs consider the simple example of region where a particular SNP always occurs in a specific haplotype background. Further, assume that the SNP has a frequency of 10% and that the haplotype has a frequency of 20%. In this example, whenever we observed this particular haplotype we expect the SNP will be present ∼50% of the time so that >50% imputation accuracy cannot be achieved for this SNP. On the other hand, knowledge of the haplotype does provide useful information about the SNP, and the rsq_hat statistic takes a value of about 0.44 in this setting. There are examples of association between imputed SNPs with similar rsq_hat statistics and complex traits that have been confirmed in follow-up genotyping (e.g. the association between *CETP* and HDL cholesterol) [16].

The studies analysed with IMPUTE typically used an additional filter to exclude imputed genotypes with a posterior probability <0.9 for IMPUTE. The proper_info measurement is interpretable as a value of x (between 0 and 1) means (approximately) that the amount of statistical information (about the parameter of interest in the model) at the imputed SNP is equivalent to perfect genotype data in a sample 100×% as big as the used sample size.

Empirical assessments show that genotype imputation using either MACH or IMPUTE provides an effective and accurate means of evaluating evidence for association at untyped markers [Li et al, Ann Rev Genom Hum Genet, *in press*; [35]–[37]. Furthermore, imputation is typically accurate even for strongly associated markers (see the Text S1 [16],[38]).

#### Study-specific stage 1 GWAS analysis

The GWAS analysis was performed by each study applying a standardized phenotype transformation to WC or WHR, respectively. Subjects were stratified by gender and the gender-specific rank of either the raw phenotype data or the residuals of a linear regression of the raw phenotype on age and age^2^ were inverse-normal transformed to yield normally distributed phenotypes. In case-control studies, cases and controls were analyzed separately. The additive genetic effect for each genotyped or imputed SNP was estimated using a linear regression model. For studies where the inverse-normal transformation was performed on raw phenotypes rather than residuals of the phenotype, age and age^2^ covariates were included in these tests. Some studies used association testing which takes genotype and imputation uncertainty into account using a missing data likelihood test implemented using SNPTEST [34] (http://www.stats.ox.ac.uk/~marchini/software) or by using allele dosages as the independent variable in the linear regression model in MACH2QTL [33] (http://www.sph.umich.edu/csg/abecasis/MACH/download/). To account for the clear gender dimorphism in these traits, analyses were performed in men and women separately, apart from the SardiNIA and deCODE studies for which, due to family relatedness between men and women, a combined analysis of men and women together was also performed, which accounted both for relatedness and gender in a single test [12]. For these GWAS analyses the software packages MACH2QTL [33], Merlin [39] and SNPTEST [34] were used.

#### Meta-analysis of GWAS results

Results from the genome-wide analyses were meta-analysed by combining the separate results for men and women (or the combined gender results in the case of SardiNIA and deCODE) from each study together into one meta-analysis of overall effect for each phenotype (*mixed-gender analysis*).

Two methods were used in parallel for stage 1 meta-analyses. The first analysis approach we used was the weighted Z-method, which allows P-values and direction of effect to be combined independently of β-estimates, allowing for incompatibility between phenotype units as in the Fisher method [40], but with improved power and precision over Fisher's test [41]. In this approach, study-specific P-values and direction of effect are converted into a signed Z-statistic. These Z-statistics are then summed with weights proportional to the square root of the sample size for each study. The other approach we used for our GWAS meta-analysis combines study-specific β-estimates using the fixed effect model [42], using the inverse of the variance of the study-specific β-estimates to weight the contribution of each study. Both meta-analysis methods are implemented in METAL (www.sph.umich.edu/csg/abecasis/metal). Results from the two approaches were highly congruent in terms of P-values. The P-values we report here are those derived from the former meta-analysis method, as it was the first we performed and results using this method were used for the selection of the SNPs taken forward to follow-up. However, measures of effect size (as β and standard error (SE)) can only be obtained from the latter.

Before performing either of these methods for a phenotype, within-study genomic control (GC) correction was applied to Z-statistics and to the variance of β-estimates using lambda factors specific to each study calculated separately for each gender (*within-study GC correction*) [18]. The GC-correction approach is based on the lambda factor, which is computed as the median of all genome-wide observed test statistics (chi-square statistic) divided by the expected median of the test statistic under the null hypothesis of no association (making the assumption that the number of true associations is very small compared to the millions of tests performed). For each study-gender combination, the observed test statistic at each SNP was divided by the lambda factor to obtain GC corrected results.

After performing each meta-analysis, we again calculated a lambda factor based on the distribution of overall test statistics, i.e. from β-estimates and SE based on the fixed effect method or on the Z-score from the weighted Z-score method. The overall lambda factors in the mixed-gender analysis were λ_GC_WC_ = 1.09 and λ_GC_WHR_ = 1.07 for the waist phenotypes. GC correction based on these overall lambda factors was then also applied (*overall GC correction*). We also found it informative to calculate λ_GC_1000_, which is an adjusted inflation factor for an equivalent study of 1,000 cases and 1,000 controls which can be calculated by rescaling λ_GC_ as previously described [19]. The results for the mixed-gender analysis for these adjusted inflation factors were λ_GC_WC_1000_ = 1.003 and λ_GC_WHR_1000_ = 1.002 for the two waist phenotypes.

Finally, we computed (based on β-estimates and SE) I^2^ statistics and Q-statistic P-values as measures of observed heterogeneity (Table S2).

#### SNP selection for replication

We aimed to select SNPs for replication that were enriched for signals of association with measures of central adiposity relative to overall adiposity or body size. To this end, we based our selection of markers for replication on evidence of association with WC or WHR (measures of central adiposity and fat distribution), while attempting to avoid associations that were primarily driven by associations with BMI (as an index of overall adiposity).

First, we identified SNPs with a P-value≤10^−5^ in the preliminary analyses with at least one of the phenotypes. This set of SNPs was then separated into independent loci by taking the most significantly associated SNP and eliminating all SNPs that have a HapMap CEU pair-wise correlation coefficient (r^2^)>0.2 with that SNP, then proceeding to the next strongly associated SNP remaining. Seventy-six independent loci each represented by one main SNP met these criteria in our preliminary analysis.

Previous experience with genome-wide association studies of anthropometric traits such as BMI [10], [14], [16]–[17],[20] and height [43]–[46] suggested that large numbers of additional samples would be required to establish association at levels of genome-wide significance. We focused our attention on 23 SNPs that showed strong association with at least one of the waist phenotypes, but with less significant evidence of association with BMI (p>0.01) and height (p>0.005), from previous analyses performed of stage 1 GWAS data within the GIANT consortia. These 23 SNPs thus had significantly stronger evidence of association with the waist phenotypes in our initial genome wide meta-analysis data than with BMI or height in previous meta-analyses involving comparable numbers of subjects.

We also added three SNPs that, despite not meeting all the P-value selection criteria, were near the borderline (Table S2) and for which biological credentials warranted selection:

1. rs7970350, which maps very near the *HMGA2* (12q15) gene. In addition to being a strong biological candidate for height, *HMGA2* is a strong biological candidate for obesity; rare mutations in this gene have previously been shown to alter body size in mice and humans. Hmga2−/− mice have a deficiency in fat tissue and resist diet-induced obesity [47]. Furthermore, the expression of a truncated *HMGA2* gene induces gigantism associated with lipomatosis [48]. This marker is in perfect linkage disequilibrium (LD) (r^2^ = 1) with a previously described locus for height (rs1042725) [43],[45],[46]. Given the low correlation between waist-circumference and height, as well as the obvious candidacy for both height and obesity, we hypothesized that this loci might affect body shape (i.e. with independent effects on height and obesity).
2. rs11970116, which maps ∼90 kb upstream of the hypocretin (orexin) receptor 2 gene, *HCRTR2* (6p11-q11). Orexins and their receptors are good candidate genes for adiposity as the orexin pathway has been implicated in the control of energy homeostasis as well as in narcolepsy and sleep patterns (ref 21). It has also been reported that hypothalamic orexin promotes appetite and that HCRTR2 signaling confers resistance to diet-induced features of the metabolic syndrome through negative energy homeostasis and improved leptin sensitivity [49]–[51].
3. rs987237, which maps to intron 3 of the *TFAP2B* (6p12) gene to add a second SNP in the vicinity of this locus in addition to rs4715215 that was already selected as one of our 23 SNPs for follow-up (pair wise r^2^ = 0.236; D′ = 1). While rs4715215 is located ∼145 kb downstream of *TFAP2B*, rs987237 is located within the gene transcript (Figure 2).

Thus, including both the 23 SNPs meeting our filtering criteria and the three additional variants, we targeted a total of 26 independent SNPs for replication in additional samples. As there were some SNPs for which the stage 1 association met the selection criteria for more than one of the waist phenotypes, there were 30 analyses to be performed (see Tables S2 and S6).

### Follow-Up in Independent GIANT Consortium Samples (GIANT Stage 2)

#### Studies and phenotypes

For our own Stage 2 analysis, we sought follow-up samples from two independent routes: we included studies with pre-existing GWAS *in-silico* data (stage 2a) as well as *de novo* genotyping (stage 2b) comprising 27 cohorts for WC and 21 cohorts for WHR. Among these stage 2 studies, 18 studies were also able to provide data on BMI, weight, and height. All individuals included in stage 2 studies were of European ancestry and provided informed consent. All studies were approved by the local ethics committees. Study-specific information on study design and participants, phenotype means, and experimental detail for all stage 2 studies are included in Tables S3, S4, S5.

#### Additional phenotypes

In addition to data on the waist phenotypes (WC and WHR) and other relevant anthropometric traits (BMI, weight, and height), we also had some cohorts from both stage 1 and stage 2 which were able to provide bioimpedance data (BIA) and/or Dual energy X-ray absorptiometry (DXA). In stage 1, three studies were informative for BIA (maximum N = 9,852), and two (maximum N = 2,308) had data on DXA. In stage 2, a total of seven cohorts had BIA (maximum N = 20,934) and six had DXA data (maximum N = 12,954). Thus, the total sample size for BIA and DXA was 30,786 or 15,262, respectively.

#### Genotypes

Genotypes were obtained from stage 2a studies, in which each SNP was either directly genotyped or imputed from genome-wide data using the CEU HapMap reference panel, and from stage 2b using *de novo* genotyping undertaken using a variety of platforms including Biotrove, Centaurus, KASPar, Sequenom, Sequenom iPLEX, and TaqMan-based assays.

Genotyping platforms, calling algorithms, quality control before imputation, imputation methods, and analysis software used were all study-specific (see Table S5 for detailed information on each study). The explicit number of follow-up SNPs genotyped in each study and whether a proxy SNP was used is summarized in Table S6.

#### Study-specific stage 2 association analyses

To analyze the two waist phenotypes in the stage 2 studies, we used the same analysis model as in stage 1 (inverse-normal transformed WC or WHR adjusted for age and age^2^ analyzed in a linear regression, all performed separately in men and women).

Additional analyses were performed - all separately in men and women and all using an additive genetic effect model - to obtain:

- waist phenotype association independent from overall obesity (using inverse-normal transformed WC or WHR adjusted for age, age^2^ and BMI)
- raw estimates of effect sizes for WC and WHR (using untransformed WC or WHR, adjusted for age and age^2^)
- raw estimates of BMI effect sizes (using untransformed BMI adjusted for age and age^2^)
- association estimates in studies with % fat phenotypes (using untransformed % total fat BIA, % total fat DXA, % central fat DXA adjusted for age and age^2^).

#### GIANT Stage 2 meta-analyses

We performed a meta-analysis for the phenotypes of primary interest (WC and WHR) of all stage 2 studies using the same methods as in stage 1 (pooled P-values using the weighted Z-score method; pooled β- and SE estimates using the fixed effect method; as well as heterogeneity statistics).

#### Meta-analysis of all GIANT data (stage 1+stage 2)

We combined GIANT stage 1 and stage 2 samples to derive a combined meta-analysis of all studies, performed in the same manner as in stage 1 and stage 2 analyses. Results from stage 1 and stage 2 studies were combined into one N-way meta-analysis.

Five of the 26 loci that were selected for follow up show nominal evidence of association with both WC and WHR (*TFAP2B* (rs987237) was one of these). However, for none of these loci did the association with WHR reach genome-wide significance in the overall, combined analysis (Table S2).

#### GIANT Gender-specific meta-analysis (stage 1, stage 2, and stage 1+2)

The waist phenotypes exhibit strong gender-differences and evidence for some genetic effects on fat distribution [7], so we performed additional meta-analyses of our stage 1 GWAS in which men and women were analyzed separately. We also tested whether the effect estimate resulting from the gender-specific fixed effect meta-analysis differed significantly between men and women by applying a t-test comparing β-effect and SE estimates in men with the β-effect and SE estimates in women. The gender-specific meta-analyses were performed on stage 1, stage 2, and combined stage 1+2 data.

### Additional Replication through Further Follow-Up Using *In Silico* Results from the CHARGE Consortium

Further, our genome wide signals for WC identified after stage 2 were confirmed using data from the “Cohorts for Heart and Aging Research in Genomic Epidemiology” (CHARGE) consortium, which members had performed a GWAS meta-analysis of 31,375 samples for WC (Table 1).

#### Studies, phenotypes, and genotypes

The CHARGE consortium consisted of 31,375 individuals from 8 studies informative for WC, though two studies overlapped with our stage 2a studies (the Erasmus Rucphen Family Study (ERF) and the Rotterdam Study (ERGO), (up to 6,702 individuals) which were included in both CHARGE and stage 2 data, but which are counted only once in the overall meta-analysis.

#### Meta-analysis of stage 1+2 results with CHARGE data

We combined the association results for WC from the GIANT and CHARGE samples to derive a combined meta-analysis of all studies (Figure 1). This analysis was performed using the METAL software for pooling of the P-values based on the weighted Z-score method, using the P-values calculated in our stage 1+2 meta-analysis (excluding ERF and ERGO) along with the P-values from CHARGE. For a more detailed description of the CHARGE consortium studies and their analysis methods, ([Fox et al. submitted to PLOS Genetics (2008)] and [http://web.chargeconsortium.com/]).

For the *MSRA* locus, genotypes for rs7826222 were only available for a subset of the CHARGE samples (N = 8,097). This is due to the fact that this SNP has been renamed to rs545854 in NCBI build 36 and was consequently one of the SNPs omitted from HapMap release 22 and therefore is not present in build 36 imputations based on that release of HapMap. Nonetheless, the effect of rs7826222 in CHARGE was directionally-consistent (P = 0.28), and CHARGE data available in larger sample size (N = 31,372) for two moderately-good proxies (rs1876511 and rs613080, both r^2^ = 0.76 with rs7826222/rs545854) and both show some support (both had directionally-consistent effect-sizes and P = 0.078) with the other findings.

## Supporting Information

Table S1 Phenotypic correlation between anthropometric values in men and women.
(0.02 MB XLS)

Table S2 The loci that were followed up in GIANT Stage 2.
(0.04 MB XLS)

Table S3 Description of samples included in the GWA meta-analysis (Stage 1) and follow-up studies (stage 2).
(0.03 MB XLS)

Table S4 Descriptive characteristics of genome-wide association study cohorts (Stage 1) and follow-up study cohorts (Stage 2).
(0.04 MB XLS)

Table S5 Information on genotyping methods, quality control of SNPs, imputation, and statistical analysis for Stage 1 and 2 study cohorts.
(0.04 MB XLS)

Table S6 Lead markers and proxies used in stage 2 replication.
(0.03 MB XLS)

Table S7 Association analysis of fat phenotypes using raw estimates for the significant loci.
(0.02 MB XLS)

Table S8 The effect of the genome wide significant adiposity loci on lipids within ENGAGE cohorts.
(0.02 MB XLS)

Table S9 The effect of the genome wide significant adiposity loci on type 2 diabetes risk.
(0.02 MB XLS)

Table S10 The association results for waist-circumference and waist-hip-ratio to the recently reported 17 BMI loci.
(0.03 MB XLS)

Text S1 Supporting text and acknowledgments.
(0.16 MB DOC)
