# Correction: Genome-Wide Association Scan Meta-Analysis Identifies Three Loci Influencing Adiposity and Fat Distribution

**DOI:** 10.1371/annotation/b6e8f9f6-2496-4a40-b0e3-e1d1390c1928

**Published:** 2009-07-27

**Authors:** Cecilia M. Lindgren, Iris M. Heid, Joshua C. Randall, Claudia Lamina, Valgerdur Steinthorsdottir, Lu Qi, Elizabeth K. Speliotes, Gudmar Thorleifsson, Cristen J. Willer, Blanca M. Herrera, Anne U. Jackson, Noha Lim, Paul Scheet, Nicole Soranzo, Najaf Amin, Yurii S. Aulchenko, John C. Chambers, Alexander Drong, Jian'an Luan, Helen N. Lyon, Fernando Rivadeneira, Serena Sanna, Nicholas J. Timpson, M. Carola Zillikens, Jing Hua Zhao, Peter Almgren, Stefania Bandinelli, Amanda J. Bennett, Richard N. Bergman, Lori L. Bonnycastle, Suzannah J. Bumpstead, Stephen J. Chanock, Lynn Cherkas, Peter Chines, Lachlan Coin, Cyrus Cooper, Gabriel Crawford, Angela Doering, Anna Dominiczak, Alex S. F. Doney, Shah Ebrahim, Paul Elliott, Michael R. Erdos, Karol Estrada, Luigi Ferrucci, Guido Fischer, Nita G. Forouhi, Christian Gieger, Harald Grallert, Christopher J. Groves, Scott Grundy, Candace Guiducci, David Hadley, Anders Hamsten, Aki S. Havulinna, Albert Hofman, Rolf Holle, John W. Holloway, Thomas Illig, Bo Isomaa, Leonie C. Jacobs, Karen Jameson, Pekka Jousilahti, Fredrik Karpe, Johanna Kuusisto, Jaana Laitinen, G. Mark Lathrop, Debbie A. Lawlor, Massimo Mangino, Wendy L. McArdle, Thomas Meitinger, Mario A. Morken, Andrew P. Morris, Patricia Munroe, Narisu Narisu, Anna Nordström, Peter Nordström, Ben A. Oostra, Colin N. A. Palmer, Felicity Payne, John F. Peden, Inga Prokopenko, Frida Renström, Aimo Ruokonen, Veikko Salomaa, Manjinder S. Sandhu, Laura J. Scott, Angelo Scuteri, Kaisa Silander, Kijoung Song, Xin Yuan, Heather M. Stringham, Amy J. Swift, Tiinamaija Tuomi, Manuela Uda, Peter Vollenweider, Gerard Waeber, Chris Wallace, G. Bragi Walters, Michael N. Weedon, The Wellcome Trust Case Control Consortium, Jacqueline C. M. Witteman, Cuilin Zhang, Weihua Zhang, Mark J. Caulfield, Francis S. Collins, George Davey Smith, Ian N. M. Day, Paul W. Franks, Andrew T. Hattersley, Frank B. Hu, Marjo-Riitta Jarvelin, Augustine Kong, Jaspal S. Kooner, Markku Laakso, Edward Lakatta, Vincent Mooser, Andrew D. Morris, Leena Peltonen, Nilesh J. Samani, Timothy D. Spector, David P. Strachan, Toshiko Tanaka, Jaakko Tuomilehto, André G. Uitterlinden, Cornelia M. van Duijn, Nicholas J. Wareham, Hugh Watkins for the PROCARDIS consortia, Dawn M. Waterworth, Michael Boehnke, Panos Deloukas, Leif Groop, David J. Hunter, Unnur Thorsteinsdottir, David Schlessinger, H.-Erich Wichmann, Timothy M. Frayling, Gonçalo R. Abecasis, Joel N. Hirschhorn, Ruth J. F. Loos, Kari Stefansson, Karen L. Mohlke, Inês Barroso, Mark I. McCarthy for the GIANT consortium

The affiliation for 65th author Johanna Kuusisto and 115th author Markku Laakso was incorrect. The correct affiliation is: Department of Medicine, University of Kuopio and Kuopio University Hospital, Kuopio, Finland.

