# Supplementary material for: Genome-Wide Association Scan Meta-Analysis Identifies Three Loci Influencing Adiposity and Fat Distribution
Source: PLoS Genet. 2009 Jun 26;5(6):e1000508. doi: 10.1371/journal.pgen.1000508 (PMC2695778; doi:10.1371/journal.pgen.1000508)
Supplement: Text S1 — Supporting text and acknowledgments. (0.16 MB DOC) [file pgen.1000508.s011.doc]

# ADDITIONAL RESULTS

## Meta-analysis of waist phenotypes in GIANT stage 1 and stage 2 studies

**Stage 1+2:** After combining the data from the original meta-analysis (stage 1), the *in-silico* follow-up (stage 2a), and the *de novo* genotyping (stage 2b), three loci attained genome-wide significance in the combined analysis (p<5x10^-8^; see Table 1 or Table S2). Further, two additional loci showed strong but less compelling evidence for association (P<5x10^-7^) with waist phenotypes. These included SNPs mapping near to the genes encoding high mobility group AT-hook 2 (*H, P=MGA2)* on chromosome 12q14.3 (rs7970350, WC, P=2.2x10^-7^) and platelet-derived growth factor receptor-like (*PDGFRL)* on chromosome 8p22-p21.3 (rs2245667, WC, P=3.1x10^-7^) (see Table S2). The role of these *genes* on central adiposity phenotypes will require further study.

## Association of waist phenotypes independent from BMI

As we had a specific interest whether the three replicating loci identified through analysis of waist phenotypes were independent from BMI or height, we first examined the relationships between the phenotypes (Table S1). Noteworthy is that WC and WHR are largely independent from height and thus we did not consider height further in our follow-up analysis.

We checked all our significant associations for the waist phenotypes adjusted not only for age and age², but also for BMI and we also analyzed the SNPs for BMI to see whether we could distinguish between central adiposity and overall adiposity. At the *TFAP2B* locus rs987237 we noticed a very strong association with BMI (P=7.0x10^-12^) in our combined stage 2 data. Further, when correcting the WC (P=1.5x10^‑5^) associations for BMI, the evidence for association was markedly reduced (P=0.03, N=53,390, stage 2).

The genetic variant close to *MSRA* (rs7826222) showed a slightly weaker association (P=2.2x10^-3^, N=57,303) with BMI as compared to the WC association (P=4.3x10^-4^, N=35,230) in stage 2 and when correcting for BMI the WC association was largely reduced (P=0.12, N=35,247).

Interestingly, the effect of the gender-specific SNP close to *LYPLAL1* (rs2605100) was associated with an BMI decreasing effect (stage 2 women, P=1.88x10^-4^, N=36,514). In the analysis of WHR adjusted for BMI, the association was accordingly stronger (stage 2 women, P=4.3x10^-6^, N=25,405) than for WHR without the adjustment for BMI.

## Association of replicating SNPs for fat phenotypes

These analyses include information on between 29,316 individuals with BIA % total fat measures for the three significant signals derived from our own stage 2 analyses (Table S7). Each of these showed at least a tendency for association with BIA %total fat with P-values ranging from 0.02 - 0.08 and the effect in BIA %total fat ranging from 0.15%-0.75%. The %total fat DXA were consistent in direction and comparably strongly associated with regard to the P-value, except for the *MSRA* SNP, for which the sample size was much lower for DXA. Of the 13,039 individuals with DXA measures of overall fat-mass, only up to 7,346 individuals had specific data on central fat mass (%central fat) (Table S7). These analyses were therefore markedly less well powered than the other analyses reported herein. Further studies in large, well-phenotyped samples will be needed in order to draw any conclusions about the effect of these loci on %central fat.

***DESCRIPTION OF THE SIGNIFICANTLY ASSOCIATED LOCI***

“Upstream” refers to the distance from the SNP to the start of the first exon and “downstream” refers to the distance from the SNP to the end of the last exon of the largest refseq transcript. All positions, SNPs and genes referred to are on Human May 2004 Assembly (Build 35.1, dbSNP build 125). To see in which tissues the genes in the associated loci (main text Table 1, Figure 2) were expressed we queried BioGPS ([https://biogps.gnf.org](https://biogps.gnf.org/)).

**1) Region near *LYPLAL1* (1q41):** rs2605100 is located in a gene-desert region on chromosome 1q41 (216,032,619 bp) 138,724 bp downstream of hypothetical protein LOC388739 (216,171,343-216,174,806 bp) and 259,066 bp up-streams of lysophospholipase-like 1 (*LYPLAL1*) (215,735,618-215,773,553bp). *LYPLAL1 is* thought to act as a triglyceride lipase that has been reported to be up-regulated in the subcutaneous and visceral adipose tissue of obese subjects[1]. *LYPLAL1* is reported in public databases to be expressed in thymus and blood cells, but also adipocytes, kidney and pancreas ([https://biogps.gnf.org/#goto=genereport&id=127018](https://biogps.gnf.org/#goto=genereport&id=127018)).

**2) Region of the *TFAP2B* locus (6p12.3):** rs987237 (50,911,009 bp) is located on chromosome 6p12.3, in the third intron of *TFAP2B* (50,894,542-50,919,634 bp). This gene encodes a member of the AP-2 family of transcription factors. AP-2 proteins form homo- or hetero-dimers with other AP-2 family members and bind specific DNA sequences. They are thought to stimulate cell proliferation and suppress terminal differentiation of specific cell types during embryonic development. TFAP2B functions as both a transcriptional activator and repressor. Mutations in this gene result in autosomal dominant Char syndrome, suggesting that this gene functions in the differentiation of neural crest cell derivatives [2]. An association between non-HapMap variants in intron 1 of *TFAP2B* and T2D has previously been reported in Japanese [3]. *TFAP2B* is reported to be preferentially expressed in adipose tissue [4], and over-expression in 3T3L1 adipocytes leads to decreased insulin sensitivity via enhanced glucose transport and increased lipid accumulation [4]. Over-expression of *TFAP2B* also down-regulates expression of the insulin-sensitizing hormone adiponectin by direct transcriptional repression [5]. Genetic variants within *TFAP2B* have recently been reported to be functional and to positively correlate with *TFAP2B* transcript levels in adipose tissue[4]. In public databases *TFAP2B*, is expressed in both adipose tissue, skeletal muscle and hypothalamus ([https://biogps.gnf.org/#goto=genereport&id=7021](https://biogps.gnf.org/#goto=genereport&id=7021)).

# 3) Region near *MSRA* (8p23.1): rs7826222 is located on chromosome 8p23.1 (9,897,490 bp) 51,746 bp upstream from *MSRA* (9,949,236-10,323,803 bp). MSRA (methionine sulfoxide reductase A) encodes an antioxidant repair enzyme that reduces oxidized methionine to methionine. Moreover, the oxidation of methionine residues in proteins is considered to be an important consequence of oxidative damage to cells[6]. Oxidation of proteins by reactive oxygen species is associated with aging, oxidative stress, and many diseases and a mutant mouse that lacks the *Msra* gene, compared with the wild type, exhibited enhanced sensitivity to oxidative stress[7]. Excess lipid accumulation leads to increased ER activity, which ultimately can overwhelm the capacity of the ER to properly fold nascent proteins. If this process proceeds unchecked, apoptosis may result. ER stress can lead to oxidative stress in the mitochondrion, as does the presence of excess free fatty acids (FFA) which through downstream mechanisms can contribute to cellular insulin resistance [8]. In public databases *MSRA* is predominantly expressed in kidney followed closely by the liver but also in brain and adipose tissue (**[https://biogps.gnf.org/#goto=genereport&id=4482](https://biogps.gnf.org/#goto=genereport&id=4482)**). An alternative candidate in the vicinity is *TNKS,* which encodes a TRF1-interacting ankyrin-related ADP-ribose polymerase (tankyrase). Tankyrase is a peripheral membrane protein known to interact with insulin-responsive aminopeptidase (IRAP) in GLUT4 vesicles in adipocytes [9,10]. Thus *TNKS* has a putative role in insulin-regulated glucose disposal into fat and other tissues. In public databases *TNKS* appear to be ubiquitously expressed in a large number of tissues (http://biogps.gnf.org/#goto=genereport&id=8658).

**Detailed Acknowledgements**

The authors would like to thank the many colleagues who contributed to collection and phenotypic characterization of the clinical samples, as well as genotyping and analysis of the GWAS data. They would also like to acknowledge those who agreed to participate in these studies. Major funding for the work described in this paper comes from:

**The British 1958 BC:** We acknowledge use of genotype data from the British 1958 Birth Cohort DNA collection, funded by the Medical Research Council grant G0000934 and the Wellcome Trust grant 068545/Z/02

**WTCCC-T2D:** Collection of the type 2 diabetes cases was supported by Diabetes UK, BDA Research and the UK Medical Research Council (MRC) (Biomedical Collections Strategic Grant G0000649). The UK Type 2 Diabetes Genetics Consortium collection was supported by the Wellcome Trust (Biomedical Collections Grant GR072960). The UK GWAS genotyping was supported by the Wellcome Trust (076113), and replication genotyping was supported by the Wellcome Trust (076113), MRC (G0601261), Diabetes UK, European Commission (EURODIA LSHG-CT-2004-518153) and the Peninsula Medical School. Personal funding comes from the Wellcome Trust (A.T.H.; Research Leave Fellow; Research Career Development Fellow); Diabetes UK (R.M.F.). M.N.W. is Vandervell Foundation Research Fellow at the Peninsula Medical School. C.M.L. is a Wellcome Trust Research Career Development Fellow (086596). We acknowledge the assistance of many colleagues involved in sample collection, phenotyping and DNA extraction in all the different studies. We thank K. Parnell, C. Kimber, A. Murray and K. Northstone for technical assistance. We thank S. Howell, M. Murphy and A. Wilson (Diabetes UK) for their long-term support for these studies. Finally, we acknowledge all participants in the various studies. CNAP and AM are supported by the Scottish Executive Chief Scientist’s Office as part of the Generation Scotland initiative.

**KORA 3 & 4:** The authors acknowledge the National Genome Research Net Germany and the Munich Center of Health Sciences as part of LMUinnovativ

**Twins UK:** The authors acknowledge financial support from the Wellcome Trust, the Department of Health via the National Institute for Health Research (NIHR) comprehensive Biomedical Research Centre award to Guy's & St Thomas' NHS Foundation Trust in partnership with King's College London/ Arthritis Research Campaign/ EC FP6 Programme Grant-512066 (LSHG-CT-2004)MOLPAGE // EC Framework 7 Health-2007-A ENGAGE ) / Biotechnology and biological Sciences Research Council (BBSRC). NS is funded by the Wellcome Trust and FP-6 (LSHM-CT-2006-037197). We thank the study participants and staff from the TwinsUK studies, in particular Irina Gillham-Nasenya, Gail Clement; staff of the DNA Collections and Genotyping Facilities at the Wellcome Trust Sanger Institute for sample preparation, genotyping and quality control, including Mike Inouye, Simon Potter, Vasudev Kumanduri, Rhian Gwilliam, Pam Whittaker, and Radhi Ravindrarajah; Le Centre National de Génotypage, France, led by Mark Lathrop, for genotyping; Duke University, North Carolina, USA, led by David Goldstein, for genotyping; and the Finnish Institute of Molecular Medicine, Finnish Genome Center, University of Helsinki, led by Aarno Palotie.

**The EPIC Study:** The EPIC Norfolk Study is funded by Cancer Research United Kingdom and the Medical Research Council. I.B. acknowledges support from EU FP6 funding (contract no LSHM-CT-2003-503041) and by the Wellcome Trust.

**INCHIANTI:** A portion of that support was through a R&D contract with MedStar Research Institute, the Intramural Research Program of the NIH, National Institute on Aging.

**GEMS:** The authors acknowledge Philip Barter, Gerard Waeber, Ruth McPherson, Robert Mahley, Tom Bersot, Antero Kesaniemi, Jonathan Cohen

**Fenland:** The Fenland Study is funded by the Wellcome Trust and the Medical Research Council, as well as by the Support for Science Funding programme and CamStrad. We are grateful to all the volunteers for their time and help, and to the General Practitioners and practice staff for help with recruitment. We thank the Fenland Study co-ordination team and the Field Epidemiology team of the MRC Epidemiology Unit for recruitment and clinical testing.

**Umea:** The authors acknowledge Göran Hallmans, Åsa Agren, Kerstin Enqvist, Thore Johnsson, Västerbottens Intervention Project, and the Sanger genotyping staff. Part of this work was supported in by grants from Novo Nordisk (370579201 to PWF); the Swedish Heart-Lung Foundation (20070633 to PWF) and the Swedish Diabetes Association (DIA2006-013 to PWF). PWF was in part supported by funding from Västerbotten’s Health Authority (ALF strategic appointment 2006-2009).

**Northern Finland Birth Cohort 1966:** The work on the Northern Finland Birth Cohort 1966 study was supported by the Academy of Finland (104781), MRC (G0500539), and the Wellcome Trust (Project Grant GR069224), Biocenter University of Oulu, Finland, STAMPEED and ENGAGE. The DNA extractions, sample quality controls, biobank up-keeping and aliquotting was performed in the national Public Health Institute, Biomedicum Helsinki, Finland and supported by the grants to Dr. Leena Peltonen from the Academy of Finland and Biocentrum Helsinki. We appreciate the help of Outi Tornwall and Minttu Jussila in DNA biobanking.

**WTCCC-CAD:** Collection of the CAD used in the WTCCC was funded by the British Heart Foundation and the Medical Research Council. N.J.S. hold a BHF Chair.

**The Herefordshire Study:** The Hertfordshire Cohort Study was funded through support from the MRC and the Arthritis Research Campaign.

**MRC-Ely:** The MRC Ely Study was funded by the Medical Research Council and the Wellcome Trust

**Rotterdam-ERGO:** The Rotterdam study was funded by the Netherlands Organization for Scientific Research under the Research Institute for Diseases in the Elderly (grant 014-90-001) and the European Commission (QL46-CT-2002–02629, GENOMOS) and is supported by the by the Netherlands Genomics Initiative (NGI)/Netherlands Organisation for Scientific Research (NWO) project nr. 050-060-810.

**ERF (Rotterdam):** The ERF study was supported by a joint grant the Center of Medical Systems Biology (CMSB).

**FUSION & METSIM:** The FUSION and METSIM studies thank the Finnish citizens who generously participated in these studies. Support for FUSION was provided by NIH grants DK062370 (MB) and DK072193 (KLM), intramural project number 1Z01 HG000024 (FSC), and a postdoctoral fellowship award from the American Diabetes Association (CJW). Genome-wide genotyping was performed by the Johns Hopkins University Genetic Resources Core Facility (GRCF) SNP Center at the Center for Inherited Disease Research (CIDR) with support from CIDR NIH Contract Number N01-HG-65403. Support for METSIM was provided by grant 124243 from the Academy of Finland (ML).

**NHS:** We thank Constance Chen for data management and analysis. We thank the

participants in the Nurses' Health Studies. The Nurses' Health Studies are supported

by US NIH grants CA65725, CA87969, CA49449, CA67262, CA50385 and 5UO1CA098233. replication data on samples of the Nurses’ Health Study is funded by the Intramural Research Program of Eunice Kennedy Shriver National Institute of Child Health and Human Development

**deCODE:** deCODE authors would like to thank participants in deCODE cardiovascular- and obesity studies and collaborators for their cooperation. We would also like to acknowledge the staff at the Clinical Research Centre (Iceland) and the deCODE Genetics biological materials and genotyping facilities for their work. We thank Gunnar Sigurdsson and Unnur Styrkarsdottir for generously providing DEXA data from the deCODE osteoporosis study. The research performed at deCODE Genetics was part funded through the European Community's Seventh Framework Programme (FP7/2007-2013), ENGAGE project, grant agreement HEALTH-F4-2007- 201413.

**WTCCC-HT:** We thank the participants of the British Genetics of Hypertension (BRIGHT) Study. The BRIGHT study and current work are supported by the Medical Research Council of Great Britain (grant number; G9521010D) and the British Heart Foundation (grant number PG02/128). The Wellcome Trust Case Control Consortium was funded by the Wellcome Trust (grant number; 076113/B/04/Z). The Barts and The London Charity funded the Barts and The London Genome Centre. Profs Dominiczak and Samani are British Heart Foundation Chairholders. Chris Wallace is funded by the British Heart Foundation (grant number: FS/05/061/19501).

**CoLaus:** The CoLaus study was supported by research grants from GlaxoSmithKline, Swiss National Science Foundation (Grant 33CSCO-122661) and from the Faculty of Biology and Medicine of Lausanne, Switzerland. The authors would like to express their gratitude to the participants in the Lausanne CoLaus study, to the investigators who have contributed to the recruitment, in particular Yolande Barreau, Anne-Lise Bastian, Binasa Ramic, Martine Moranville, Martine Baumer, Marcy Sagette, Jeanne Ecoffey and Sylvie Mermoud for data collection and to Allen Roses, and Lefkos T. Middleton for their support. Some computation was carried out on the Vital-IT system at the Swiss Institute of Bioinformatics. We thank Paul Matthews, Lefkos Middleton, Dan Burns, Eric Lai and Allen Roses for their support.

**SardiNIA:** The SardiNIA project is supported by the Intramural Research Program of the NIH, National Institute on Aging. The efforts of GRA, SS and PAS were also supported in part by research grants from the National Human Genome Research Institute and the National Heart Lung and Blood Institute (Grant codes HG02651, HL084729). We would like to thank SardiNIA study participants for their volunteerism and the local civil and religious authorities in SardiNIA for their support. Gonçalo Abecasis is a Pew Scholar in the Biomedical Sciences

**BWHHS:** The British Women’s Heart and Health Study is funded by the UK Department of Health Policy Research Programme and the British Heart Foundation. NJT, GDS and DAL are supported by MRC Centre grant G0600705. The study is co-directed by Professors Shah Ebrahim, Debbie A Lawlor and Peter Whincup and Dr Goya Wannamethee. We thank Carol Bedford, Alison Emerton, Nicola Frecknall, Karen Jones, Rita Patel, Mark Taylor, Katherine Wornell, Claire Carson and Antoinette Amuzu for collecting and entering data, all of the general practitioners and their staff who have supported data collection, and the women who have participated in the study.

**UKT2DGC:** The UK Type 2 Diabetes Genetics Consortium collection was supported by the Wellcome Trust (Biomedical Collections Grant GR072960). Genotyping of the UKT2DGC and OxGN/58BC samples was supported by the European Commission (EURODIA LSHG-CT-2004- 518153), MRC (Project Grant G016121) and Diabetes UK.

**BPPP:** Genotyping and analysis in PPP-Botnia was supported by NIH grantDK075787 and an ADA Smith Family Foundation Pinnacle Program Project Award (to J.N.H.).

**ProCARDIS:** This work was funded by EC Sixth Framework Programme (LSHM-CT- 2007- 037273) and AstraZeneca AB. AH obtained support for this project from the Swedish Heart-Lung Foundation, the Swedish Medical Research Council (8691), the Knut and Alice Wallenberg Foundation, the Karolinska Institute and the Stockholm County Council (560183).

**DGI:** The studies were supported by a Linné grant from Swedish Research Council, Wallenberg Foundation, the Juselius Foundation, and the Folkhälsan Research Foundation. DGI was funded by a grant from Novartis. E.K.S. was supported by an NIH T32 DK07191 grant to Daniel K. Podolsky in the Department of Gastroenterology at Massachusetts General Hospital and NIH grants F32 DK079466 and K23 DK080145. H.N.L. was supported by NIH grant K23 DK067288.

**FINRISK97:** The Study was supported by European Community's Seventh Framework Programme grant agreement HEALTH-F4-2007- 201413, The Center Of Excellence of the Academy of Finland and the Finnish Heart Association. Genotyping in FINRISK97 was supported by NHLBI grant 5R01HL087679-02 through the STAMPEED program and NIDDK grant 5R01DK075787. P.J. and V.S. were supported by the Finish Academy grant number 118065. V.S. was also supported by a grant from the Sigrid Juselius Foundation.

**Membership of the Wellcome Trust Case Control Consortium (60. In the author list annotation):**

**Management Committee:** Paul R Burton^1^, David G Clayton^2^, Lon R Cardon^3^, Nick Craddock^4^, Panos Deloukas^5^, Audrey Duncanson^6^, Dominic P Kwiatkowski^3,5^, Mark I McCarthy^3,7^, Willem H Ouwehand^8,9^, Nilesh J Samani^10^, John A Todd^2^, Peter Donnelly (Chair)^11^

**Data and Analysis Committee:** Jeffrey C Barrett^3^, Paul R Burton^1^, Dan Davison^11^, Peter Donnelly^11^, Doug Easton^12^, David M. Evans^3^, Hin-Tak Leung^2^, Jonathan L Marchini^11^, Andrew P Morris^3^, Chris CA Spencer^11^, Martin D Tobin^1^, Lon R Cardon (Co-chair)^3^, David G Clayton (Co-chair)^2^

**UK Blood Services & University of Cambridge Controls**: Antony P Attwood^5,8^, James P Boorman^8,9^, Barbara Cant^8^, Ursula Everson^13^, Judith M Hussey^14^, Jennifer D Jolley^8^, Alexandra S Knight^8^, Kerstin Koch^8^, Elizabeth Meech^15^, Sarah Nutland^2^, Christopher V Prowse^16^, Helen E Stevens^2^, Niall C Taylor^8^, Graham R Walters^17^, Neil M Walker^2^, Nicholas A Watkins^8,9^, Thilo Winzer^8^, John A Todd^2^, Willem H Ouwehand^8,9^

**1958 Birth Cohort Controls:** Richard W Jones^18^, Wendy L McArdle^18^, Susan M Ring^18^, David P Strachan^19^, Marcus Pembrey^18,20^

**Bipolar Disorder (Aberdeen):** Gerome Breen^21^, David St Clair^21^; (**Birmingham):** Sian Caesar^22^, Katherine Gordon-Smith^22,23^, Lisa Jones^22^; **(Cardiff):** Christine Fraser^23^, Elaine K Green^23^, Detelina Grozeva^23^, Marian L Hamshere^23^, Peter A Holmans^23^, Ian R Jones^23^, George Kirov^23^, Valentina Moskvina^23^, Ivan Nikolov^23^, Michael C O’Donovan^23^, Michael J Owen^23^, Nick Craddock^23^; **(London):** David A Collier^24^, Amanda Elkin^24^, Anne Farmer^24^, Richard Williamson^24^, Peter McGuffin^24^; **(Newcastle):** Allan H Young^25^, I Nicol Ferrier^25^

**Coronary Artery Disease (Leeds):** Stephen G Ball^26^, Anthony J Balmforth^26^,

Jennifer H Barrett^26^, D Timothy Bishop^26^, Mark M Iles^26^, Azhar Maqbool^26^, Nadira Yuldasheva^26^, Alistair S Hall^26^; **(Leicester):** Peter S Braund^10^, Paul R Burton^1^, Richard J Dixon^10^, Massimo Mangino^10^, Suzanne Stevens^10^, Martin D Tobin^1^, John R Thompson^1^, Nilesh J Samani^10^

**Crohn’s Disease (Cambridge):** Francesca Bredin^27^, Mark Tremelling^27^, Miles

Parkes^27^; **(Edinburgh):** Hazel Drummond^28^, Charles W Lees^28^, Elaine R Nimmo^28^, Jack Satsangi^28^; **(London):** Sheila A Fisher^29^, Alastair Forbes^30^, Cathryn M Lewis^29^, Clive M Onnie^29^, Natalie J Prescott^29^, Jeremy Sanderson^31^, Christopher G Mathew^29^; **(Newcastle):** Jamie Barbour^32^, M Khalid Mohiuddin^32^, Catherine E Todhunter^32^, John C Mansfield^32^; **(Oxford):** Tariq Ahmad^33^, Fraser R Cummings^33^, Derek P Jewell^33^

**Hypertension (Aberdeen):** John Webster^34^; **(Cambridge):** Morris J Brown^35^, David G Clayton^2^; **(Evry, France): G** Mark Lathrop^36^; **(Glasgow):** John Connell^37^, Anna Dominiczak^37^; **(Leicester):** Nilesh J Samani^10^; **(London):** Carolina A Braga Marcano^38^, Beverley Burke^38^, Richard Dobson^38^, Johannie Gungadoo^38^, Kate L Lee^38^, Patricia B Munroe^38^, Stephen J Newhouse^38^, Abiodun Onipinla^38^, Chris Wallace^38^, Mingzhan Xue^38^, Mark Caulfield^38^; **(Oxford):** Martin Farrall^39^

**Rheumatoid Arthritis:** Anne Barton^40^, Ian N Bruce^40^, Hannah Donovan^40^, Steve Eyre^40^, Paul D Gilbert^40^, Samantha L Hider^40^, Anne M Hinks^40^, Sally L John^40^, Catherine Potter^40^, Alan J Silman^40^, Deborah PM Symmons^40^, Wendy Thomson^40^, Jane Worthington^40^

**Type ^1^ Diabetes:** David G Clayton^2^, David B Dunger^2^,^41^, Sarah Nutland^2^, Helen E Stevens^2^, Neil M Walker^2^, Barry Widmer^2^,^41^, John A Todd^2^

**Type 2 Diabetes (Exeter):** Timothy M Frayling^42^,^43^, Rachel M Freathy^42^,^43^, Hana Lango^42^,^43^, John R B Perry^42^,^43^, Beverley M Shields^43^, Michael N Weedon^42^,^43^, Andrew T Hattersley^42^,^43^; **(London):** Graham A Hitman^44^; **(Newcastle):** Mark Walker^45^; **(Oxford):** Kate S Elliott^3^,^7^, Christopher J Groves^7^, Cecilia M Lindgren^3^,^7^, Nigel W Rayner^3^,^7^, Nicholas J Timpson^3^,^46^, Eleftheria Zeggini^3^,^7^, Mark I McCarthy^3^,^7^

**Tuberculosis (Gambia):** Melanie Newport^47^, Giorgio Sirugo^47^; **(Oxford):** Emily Lyons^3^, Fredrik Vannberg^3^, Adrian VS Hill^3^

**Ankylosing Spondylitis:** Linda A Bradbury^48^, Claire Farrar^49^, Jennifer J Pointon^48^, Paul Wordsworth^49^, Matthew A Brown^48^,^49^

**AutoImmune Thyroid Disease:** Jayne A Franklyn^50^, Joanne M Heward^50^, Matthew J Simmonds^50^, Stephen CL Gough^50^

**Breast Cancer:** Sheila Seal^51^, Michael R Stratton^51^,^52^, Nazneen Rahman^51^

**Multiple Sclerosis:** Maria Ban^53^, An Goris^53^, Stephen J Sawcer^53^, Alastair

Compston^53^

**Gambian Controls (Gambia):** David Conway^47^, Muminatou Jallow^47^, Melanie

Newport^47^, Giorgio Sirugo^47^; **(Oxford):** Kirk A Rockett^3^, Dominic P Kwiatkowski^3^,^5^

**DNA, Genotyping, Data QC and Informatics (Wellcome Trust Sanger Institute, Hinxton):** Suzannah J Bumpstead^5^, Amy Chaney^5^, Kate Downes^2^,^5^, Mohammed JR Ghori^5^, Rhian Gwilliam^5^, Sarah E Hunt^5^, Michael Inouye^5^, Andrew Keniry^5^, Emma King^5^, Ralph McGinnis^5^, Simon Potter^5^, Rathi Ravindrarajah^5^, Pamela Whittaker^5^, Claire Widden^5^, David Withers^5^, Panos Deloukas^5^; **(Cambridge):** Hin- Tak Leung^2^, Sarah Nutland^2^, Helen E Stevens^2^, Neil M Walker^2^, John A Todd^2^ **Statistics (Cambridge):** Doug Easton^12^, David G Clayton^2^; **(Leicester):** Paul R Burton^1^, Martin D Tobin^1^; **(Oxford):** Jeffrey C Barrett^3^, David M Evans^3^, Andrew P Morris^3^, Lon R Cardon^3^; **(Oxford):** Niall J Cardin^11^, Dan Davison^11^, Teresa Ferreira^11^, Joanne Pereira-Gale^11^, Ingeleif B Hallgrimsdóttir^11^, Bryan N Howie^11^, Jonathan L Marchini^11^, Chris CA Spencer^11^, Zhan Su^11^, Yik Ying Teo^3^,^11^, Damjan Vukcevic^11^, Peter Donnelly^11^ **PIs:** David Bentley^5^,^54^, Matthew A Brown^48^,^49^, Lon R Cardon^3^, Mark Caulfield^38^, David G Clayton^2^, Alistair Compston^53^, Nick Craddock^23^, Panos Deloukas^5^, Peter Donnelly^11^, Martin Farrall^39^, Stephen CL Gough^50^, Alistair S Hall^26^, Andrew T Hattersley^42^,^43^, Adrian VS Hill^3^, Dominic P Kwiatkowski^3^,^5^, Christopher G Mathew^29^, Mark I McCarthy^3^,^7^, Willem H Ouwehand^8^,^9^, Miles Parkes^27^, Marcus Pembrey^18^,^20^, Nazneen Rahman^51^, Nilesh J Samani^10^, Michael R Stratton^51^,^52^, John A Todd^2^, Jane Worthington^40^

***Affiliations:*** *1 Genetic Epidemiology Group, Department of Health Sciences, University of Leicester, Adrian Building, University Road, Leicester, LE1 7RH, UK; 2 Juvenile Diabetes Research Foundation/Wellcome Trust Diabetes and Inflammation Laboratory, Department of Medical Genetics, Cambridge Institute for Medical Research, University of Cambridge, Wellcome Trust/MRC Building, Cambridge, CB2 0XY, UK; 3 Wellcome Trust Centre for Human Genetics, University of Oxford, Roosevelt Drive, Oxford OX3 7BN, UK; 4 Department of Psychological Medicine, Henry Wellcome Building, School of Medicine, Cardiff University, Heath Park, Cardiff CF14 4XN, UK; 5 The Wellcome Trust Sanger Institute, Wellcome Trust Genome Campus, Hinxton, Cambridge CB10 1SA, UK; 6 The Wellcome Trust, Gibbs Building, 215 Euston Road, London NW1 2BE, UK; 7 Oxford Centre for Diabetes, Endocrinology and Medicine, University of Oxford, Churchill Hospital, Oxford, OX3 7LJ, UK; 8 Department of Haematology, University of Cambridge, Long Road, Cambridge, CB2 2PT, UK; 9 National Health Service Blood and Transplant, Cambridge Centre, Long Road, Cambridge, CB2 2PT, UK; 10 Department of Cardiovascular Sciences, University of Leicester, Glenfield Hospital, Groby Road, Leicester, LE3 9QP, UK; 11 Department of Statistics, University of Oxford, 1 South Parks Road, Oxford OX1 3TG, UK; 12 Cancer Research UK Genetic Epidemiology Unit, Strangeways Research Laboratory, Worts Causeway, Cambridge CB1 8RN, UK; 13 National Health Service Blood and Transplant, Sheffield Centre, Longley Lane, Sheffield S5 7JN, UK; 14 National Health Service Blood and Transplant, Brentwood Centre, Crescent Drive, Brentwood, CM15 8DP, UK; 15 The Welsh Blood Service, Ely Valley Road, Talbot Green, Pontyclun, CF72 9WB, UK; 16 The Scottish National Blood Transfusion Service, Ellen’s Glen Road, Edinburgh, EH17 7QT, UK; 17 National Health Service Blood and Transplant, Southampton Centre, Coxford Road, Southampton, SO16 5AF, UK; 18 Avon Longitudinal Study of Parents and Children, University of Bristol, 24 Tyndall Avenue, Bristol, BS8 1TQ, UK; 19 Division of Community Health Services, St George’s University of London, Cranmer Terrace, London SW17 0RE, UK; 20 Institute of Child Health, University College London, 30 Guilford St, London WC1N 1EH, UK; 21 University of Aberdeen, Institute of Medical Sciences, Foresterhill, Aberdeen, AB25 2ZD, UK; 22 Department of Psychiatry, Division of Neuroscience, Birmingham University, Birmingham, B15 2QZ, UK; 23 Department of Psychological Medicine, Henry Wellcome Building, School of Medicine, Cardiff University, Heath Park, Cardiff CF14 4XN, UK; 24 SGDP, The Institute of Psychiatry, King's College London, De Crespigny Park Denmark Hill London SE5 8AF, UK; 25 School of Neurology, Neurobiology and Psychiatry, Royal Victoria Infirmary, Queen Victoria Road, Newcastle upon Tyne, NE1 4LP, UK; 26 LIGHT and LIMM Research Institutes, Faculty of Medicine and Health, University of Leeds, Leeds, LS1 3EX, UK; 27 IBD Research Group, Addenbrooke's Hospital, University of Cambridge, Cambridge, CB2 2QQ, UK; 28 Gastrointestinal Unit, School of Molecular and Clinical Medicine, University of Edinburgh, Western General Hospital, Edinburgh EH4 2XU UK; 29 Department of Medical & Molecular Genetics, King's College London School of Medicine, 8th Floor Guy's Tower, Guy's Hospital, London, SE1 9RT, UK; 30 Institute for Digestive Diseases, University College London Hospitals Trust, London, NW1 2BU, UK; 31 Department of Gastroenterology, Guy's and St Thomas' NHS Foundation Trust, London, SE1 7EH, UK; 32 Department of Gastroenterology & Hepatology, University of Newcastle upon Tyne, Royal Victoria Infirmary, Newcastle upon Tyne, NE1 4LP, UK; 33 Gastroenterology Unit, Radcliffe Infirmary, University of Oxford, Oxford, OX2 6HE, UK; 34 Medicine and Therapeutics, Aberdeen Royal Infirmary, Foresterhill, Aberdeen, Grampian AB9 2ZB, UK; 35 Clinical Pharmacology Unit and the Diabetes and Inflammation Laboratory, University of Cambridge, Addenbrookes Hospital, Hills Road, Cambridge CB2 2QQ, UK; 36 Centre National de Genotypage, 2, Rue Gaston Cremieux, Evry, Paris 91057.; 37 BHF Glasgow Cardiovascular Research Centre, University of Glasgow, 126 University Place, Glasgow, G12 8TA, UK; 38 Clinical Pharmacology and Barts and The London Genome Centre, William Harvey Research Institute, Barts and The London, Queen Mary’s School of Medicine, Charterhouse Square, London EC1M 6BQ, UK; 39 Cardiovascular Medicine, University of Oxford, Wellcome Trust Centre for Human Genetics, Roosevelt Drive, Oxford OX3 7BN, UK; 40arc Epidemiology Research Unit, University of Manchester, Stopford Building, Oxford Rd, Manchester, M13 9PT, UK; 41 Department of Paediatrics, University of Cambridge, Addenbrooke’s Hospital, Cambridge, CB2 2QQ, UK; 42 Genetics of Complex Traits, Institute of Biomedical and Clinical Science, Peninsula Medical School, Magdalen Road, Exeter EX1 2LU UK; 43 Diabetes Genetics, Institute of Biomedical and Clinical Science, Peninsula Medical School, Barrack Road, Exeter EX2 5DU UK; 44 Centre for Diabetes and Metabolic Medicine, Barts and The London, Royal London Hospital, Whitechapel, London, E1 1BB UK; 45 Diabetes Research Group, School of Clinical Medical Sciences, Newcastle University, Framlington Place, Newcastle upon Tyne NE2 4HH, UK; 46 The MRC Centre for Causal Analyses in Translational Epidemiology, Bristol University, Canynge Hall, Whiteladies Rd, Bristol BS2 8PR, UK; 47 MRC Laboratories, Fajara, The Gambia; 48 Diamantina Institute for Cancer, Immunology and Metabolic Medicine, Princess Alexandra Hospital, University of Queensland, Woolloongabba, Qld 4102, Australia; 49 Botnar Research Centre, University of Oxford, Headington, Oxford OX3 7BN, UK; 50 Department of Medicine, Division of Medical Sciences, Institute of Biomedical Research, University of Birmingham, Edgbaston, Birmingham B15 2TT, UK; 51 Section of Cancer Genetics, Institute of Cancer Research, 15 Cotswold Road, Sutton, SM2 5NG, UK; 52 Cancer Genome Project, The Wellcome Trust Sanger Institute, Wellcome Trust Genome Campus, Hinxton, Cambridge CB10 1SA, UK; 53 Department of Clinical Neurosciences, University of Cambridge, Addenbrooke’s Hospital, Hills Road, Cambridge CB2 2QQ, UK; 54 PRESENT ADDRESS: Illumina Cambridge, Chesterford Research Park, Little Chesterford, Nr Saffron Walden, Essex, CB10 1XL, UK*

**SOM References**

1. Steinberg GR, Kemp BE, Watt MJ (2007) Adipocyte triglyceride lipase expression in human obesity. Am J Physiol Endocrinol Metab 293: E958-964.

2. Satoda M, Zhao F, Diaz GA, Burn J, Goodship J, et al. (2000) Mutations in TFAP2B cause Char syndrome, a familial form of patent ductus arteriosus. Nat Genet 25: 42-46.

3. Maeda S, Tsukada S, Kanazawa A, Sekine A, Tsunoda T, et al. (2005) Genetic variations in the gene encoding TFAP2B are associated with type 2 diabetes mellitus. J Hum Genet 50: 283-292.

4. Tsukada S, Tanaka Y, Maegawa H, Kashiwagi A, Kawamori R, et al. (2006) Intronic polymorphisms within TFAP2B regulate transcriptional activity and affect adipocytokine gene expression in differentiated adipocytes. Mol Endocrinol 20: 1104-1111.

5. Ikeda K, Maegawa H, Ugi S, Tao Y, Nishio Y, et al. (2006) Transcription factor activating enhancer-binding protein-2beta. A negative regulator of adiponectin gene expression. J Biol Chem 281: 31245-31253.

6. Rahman MA, Nelson H, Weissbach H, Brot N (1992) Cloning, sequencing, and expression of the Escherichia coli peptide methionine sulfoxide reductase gene. J Biol Chem 267: 15549-15551.

7. Moskovitz J, Bar-Noy S, Williams WM, Requena J, Berlett BS, et al. (2001) Methionine sulfoxide reductase (MsrA) is a regulator of antioxidant defense and lifespan in mammals. Proc Natl Acad Sci U S A 98: 12920-12925.

8. de Ferranti S, Mozaffarian D (2008) The perfect storm: obesity, adipocyte dysfunction, and metabolic consequences. Clin Chem 54: 945-955.

9. Chi NW, Lodish HF (2000) Tankyrase is a golgi-associated mitogen-activated protein kinase substrate that interacts with IRAP in GLUT4 vesicles. J Biol Chem 275: 38437-38444.

10. Yeh TY, Sbodio JI, Tsun ZY, Luo B, Chi NW (2007) Insulin-stimulated exocytosis of GLUT4 is enhanced by IRAP and its partner tankyrase. Biochem J 402: 279-290.

11. Willer CJ, Speliotes EK, Loos RJ, Li S, Lindgren CM, et al. (2009) Six new loci associated with body mass index highlight a neuronal influence on body weight regulation. Nat Genet 41: 25-34.

12. Thorleifsson G, Walters GB, Gudbjartsson DF, Steinthorsdottir V, Sulem P, et al. (2008) Genome-wide association yields new sequence variants at seven loci that associate with measures of obesity. Nat Genet.

13. Meyre D, Delplanque J, Chevre JC, Lecoeur C, Lobbens S, et al. (2009) Genome-wide association study for early-onset and morbid adult obesity identifies three new risk loci in European populations. Nat Genet 41: 157-159.
